# Morphological Heterogeneity of the Endoplasmic Reticulum within Neurons and Its Implications in Neurodegeneration

**DOI:** 10.3390/cells10050970

**Published:** 2021-04-21

**Authors:** Sreesha Sree, Ilmari Parkkinen, Anna Their, Mikko Airavaara, Eija Jokitalo

**Affiliations:** 1 Cell and Tissue Dynamics Research Program, Institute of Biotechnology, Helsinki Institute for Life Science, University of Helsinki, Viikinkaari 9, 00014 Helsinki, Finland; Sreesha.Sree@Helsinki.fi; 2 Neuroscience Center, Helsinki Institute for Life Science, University of Helsinki, Haartmaninkatu 8, 00014 Helsinki, Finland; Ilmari.Parkkinen@helsinki.fi (I.P.); Anna.Their@Helsinki.fi (A.T.); 3 Division of Pharmacology and Pharmacotherapy, Faculty of Pharmacy, University of Helsinki, Viikinkaari 5, 00014 Helsinki, Finland; 4 Electron Microscopy Unit, Institute of Biotechnology, Helsinki Institute for Life Science, University of Helsinki, Viikinkaari 9, 00014 Helsinki, Finland

**Keywords:** neuronal endoplasmic reticulum, neurodegeneration, dopaminergic neurons, endoplasmic reticulum subdomains, sporadic neurodegeneration

## Abstract

The endoplasmic reticulum (ER) is a multipurpose organelle comprising dynamic structural subdomains, such as ER sheets and tubules, serving to maintain protein, calcium, and lipid homeostasis. In neurons, the single ER is compartmentalized with a careful segregation of the structural subdomains in somatic and neurite (axodendritic) regions. The distribution and arrangement of these ER subdomains varies between different neuronal types. Mutations in ER membrane shaping proteins and morphological changes in the ER are associated with various neurodegenerative diseases implying significance of ER morphology in maintaining neuronal integrity. Specific neurons, such as the highly arborized dopaminergic neurons, are prone to stress and neurodegeneration. Differences in morphology and functionality of ER between the neurons may account for their varied sensitivity to stress and neurodegenerative changes. In this review, we explore the neuronal ER and discuss its distinct morphological attributes and specific functions. We hypothesize that morphological heterogeneity of the ER in neurons is an important factor that accounts for their selective susceptibility to neurodegeneration.

## 1. Neuronal ER—A Historical Perspective

The first in toto illustrations of neurons were done by the founders of modern neuroscience and neuropathology, namely Ramón y Cajal, Camillo Golgi, and Franz Nissl [1,2]. Their detailed neuroanatomical studies on the perplexing morphologies of neurons, with their highly arborized extensions, have become textbook examples of neuronal morphology. Their art and illustrations were immensely accurate from what they had observed with their microscopes, however they lacked the tools and methods to see further inside the cell. The complex arrangement of organelles within neurons, especially those residing in their vast extensions thus remained unrevealed. Like in all cells, the cytoplasm in neurons is packed with organelles, and the largest organelle, the endoplasmic reticulum (ER), extends throughout the whole cell. The continuity of the ER throughout the extensive extensions of the neuron as a single organelle has even posited some to describe the structure as “a neuron-within-a-neuron” [3].

**Figure 1:**
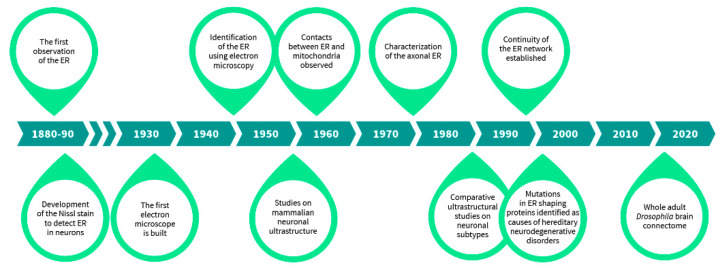
Historical timeline of research on neuronal ER.

Although the very first observations of most subcellular organelles by light microscopes were done at the end of the 19th century, mainly during the last decade, detailed characterization of different organelles was only made possible after the development of the electron microscope in 1932 by Ernst Ruska and Max Knoll (Figure 1) [4]. Originally described by Emilio Veratti in 1902 as sarcoplasmic reticulum based on his modification of Camillo Golgi’s black reaction [5], the ER was ‘rediscovered’ years later as a “lace-like reticulum” using electron microscopy by Keith Porter, Albert Claude, and Ernest Fullam in 1945 [6]. However, it was not until 1953, a year after mitochondrion was first described with electron micrographs, that the term “endoplasmic reticulum” followed by the common abbreviation, ER, was introduced by Keith Porter to describe the “lace-like” structure in the perinuclear region of the cytoplasm [6,7,8]. George Palade also observed that ribosomes were attached to the ER, which led to the distinction between rough and smooth ER, now also distinguished into sheet-like and tubular ER [9]. Even after its discovery, the 3D structure of the ER was difficult to comprehend from the available 2D graphs. It took some intuitive thinking by Fritiof Sjöstrand in 1953 to place the ER in the three-dimensional context of a living cell from the two-dimensional images [10]. The contacts between ER and mitochondria, the two major organelles, were observed in 1959 [11].

The finding of the ER, important ultrastructural studies characterizing features of the ER further, and the discovery and use of various techniques that have allowed studying the neuronal ER in its entirety.

Neuroanatomical studies were highly influenced by the development of the Nauta-Gygax method in 1954 preceding the implementation of Golgi/EM techniques in the 1960s and other techniques, which enhanced the visualization and identification of organelles by filling their lumen with electron dense material, e.g., using horseradish peroxidase, in the 1970s [12,13,14]. Studies on the neuronal ER before the era of electron microscopy relied a lot on the still widely used Nissl stain [1]. Mammalian neuronal ultrastructure was further characterized by Sanford Palay and George Palade in 1955 in their study “The fine structure of neurons”, where the relationship of the ER and Nissl bodies was carefully assessed [15]. Later, the axonal ER was largely described in 1976 by Shöichiro Tsukita and Harunori Ishikawa [16], and the continuity of the neuronal ER throughout the neuritic extensions by Mark Terasaki in 1994 [17]. Further on, specific neuronal type ultrastructure was characterized and many intriguing comparative studies, for instance on dopaminergic neurons between brain areas, were done in which organellar morphology was systematically compared [18]. These studies have been instrumental for laying out the foundation for modern diagnostic electron microscopy used in neuropathology, such as ultrastructural comparisons of schizophrenia patient substantia nigra to controls [19,20]. The latest advancements in electron microscopy, such as serial sectioning, electron tomography, and focused ion beam-scanning electron microscopy have enabled large-volume 3D reconstructions of neurons [21,22,23]. These are now widely used in connectomic studies, and a whole adult female Drosophila brain at synaptic resolution has been rendered by a custom high-throughput electron microscopy platform, effectively revealing its precise connectome [24]. Nevertheless, besides the connectomic efforts and some 3D reconstruction of specific neurons in naïve and stressed states, these techniques have not been used to their full extent to acquire detailed ultrastructural information of organelle morphologies in diseased and healthy neurons [25]. Hence, the more these techniques get incorporated and the more data we obtain on various neuronal subtype organellar architecture, we may possibly be able to link differences in morphologies to the diseases which will aid in diagnostic imaging and in the discovery of new therapeutics.

In the current review, we begin by discussing the ER morphology and the factors that contribute to shaping and maintenance of the ER architecture in general. We focus on neuronal ER and its specific attributes, discussing the significance and implications of heterogeneity in ER morphology that have been observed between different types of neurons.

## 2. General ER Morphology in Cells

**Figure 2:**
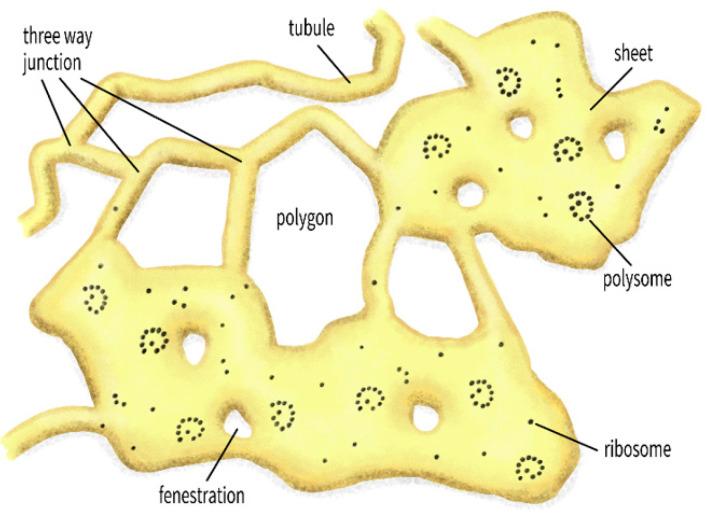
An illustration of ER network of sheets and tubules. Network polygons and branch points (called three way junctions), ribosomes, polysomes, and fenestrations on sheets are indicated.

The ER is present almost everywhere in eukaryotic cells in different morphologies, adapting itself to the varied dimensions of a cell. Some of the well-studied ER structural subdomains are the nuclear envelope—the wide ER sheets in the perinuclear area extending into the peripheral area, and the narrow cylindrical ER tubules (Figure 2). While the sheets with their attached ribosomes primarily serve as sites of protein synthesis, the tubules are generally associated with the function of lipid synthesis [9,26] with further cell type specific roles [26]. Other morphological variants seen in the ER include: Fenestrated ER sheets—ER cisternae having perforations with sealed edges [27], some transitional forms involved in the secretory pathway [28], cortical ER—the ER occurring in close proximity to the plasma membrane (PM) [29], and stacked ER sheets with each level connected by helical ramps [30]. Recently, another type of ER subdomain, the ribosome associated vesicles (RAV) of ER were identified as a common entity in multiple cell types [31]. These different ER subdomains may show additional morphological variations with specialized functions, such as the hypolemmal system in neurons [23,32]. Apart from a dedicated set of integral membrane proteins to shape and maintain them, the different morphological subdomains of the ER are equipped to cater to different functional requirements of the cell. The most intriguing part is that these different structural forms still maintain a physical continuity, thereby rendering ER the largest organelle in a eukaryotic cell [3,17]. The complex structure of the ER is also highly dynamic and changes according to the cell’s requirements. What regulates the changes is still not fully understood, but the ER network is constantly rearranging and redistributing, aided by multiple factors [33,34,35] and has even been found to be sensitive to mechanical stimuli [36].

## 3. Mechanisms Shaping the ER and ER Contact Sites in General

Factors contributing to generation and maintenance of ER framework include: (i) Membrane bending proteins and intraluminal tethering proteins, (ii) Proteins mediating homotypic ER fusion, and (iii) Cytoskeleton and motor proteins. Ribosomes or polysome attachment also contributes to stabilization of ER sheet architecture [27,37,38].

### 3.1. Role of ER Associated Proteins in Organelle Shaping

The earliest studies on ER shaping proteins were on the reticulons and their interacting partner DP1/Yop1p [39,40]. Eventually, other proteins like Receptor expression enhancing proteins (REEP), Atlastins and Spastins were identified as having a role in ER shaping too. Hitherto, several integral membrane proteins have been found to function in generating and maintaining different structural subdomains of the ER. Of these, the curvature inducing proteins exhibit a typical wedge-shaped embedding within the ER membrane. These proteins have been found to localize exclusively to ER tubules and the curved edges of the ER sheets. The creation and maintenance of sheet morphology requires factors working in a direction opposite to those required for tubule formation, stabilization of flat membrane, and maintaining constant sheet thickness. Both sheet and tubule promoting factors are regulated and work in concert to ensure an optimal shape to the ER where required. For example, the presence of membrane bending proteins like reticulons and REEPs at sheet edges are important to render the necessary curvature to ER sheets that are formed by sheet promoters such as CLIMP63 [38,41,42]. Reticulons are a ubiquitous and highly conserved family of integral membrane proteins. There are four reticulon genes, *RTN1*, *RTN2*, *RTN3*, and *RTN4*/*NOGO* known in mammals. Immunofluorescence and immuno-electron microscopy indicate the localization of reticulon proteins exclusively to ER tubules and curved edges of ER sheets [40,41]. All reticulon proteins have a conserved region called the reticulon homology domain (RHD) at their carboxy terminal. The RHD consists of two hydrophobic transmembrane (TM) regions, each of about 30–35 aa flanking a hydrophilic loop of about 66 aa (Nogo-66) length followed by a short tail region [35,43,44]. Experiments with RTN4C mutants have revealed that at least one of the TM domains adopts a hairpin shape [40]. These hairpin TM domains were found to be unusually short sized occupying more space in the outer membrane compared to the inner membrane (wedge shape) of the ER bilayer, thereby pushing the lipids sideways causing the membrane to bend [35,45]. This wedge-shaped insertion is complemented by formation of immobile oligomers by reticulons leading to formation of the stable curvature that is typical of ER tubules and sheet edges [35,39]. Overexpression of certain reticulon isoforms induces formation of tubular ER in mammalian and yeast cells, even disrupting the peripheral ER sheets in some cell types [40,41]. Depletion of reticulons led to derangement of peripheral tubular ER and formation of large peripheral ER sheets [39,40,41]. Autophagy has been widely studied in neurons and controlled recycling of various organelles, the ER included, is crucial for neuronal health and function. RTN3 and RTN-1C are involved in the turnover of the ER due to its selective autophagy, also called ER phagy [46,47,48]. Reticulons have also been recently shown to be able to constrict ER tubules which results in fragmentation of the ER, important for ER phagy [49].

REEPs are a family of six membrane bound proteins (REEP1–6) in mammals, which were first identified for their surface expression enhancing functions of selective olfactory receptors [50]. Members of the REEP family work with the reticulons to shape the ER membrane into curved tubules and edges [39,40]. The six REEPs have been divided into two subfamilies, REEP1–4 and REEP5/6 based on sequence similarities [51]. Like the reticulons, all REEPs also harbor the conserved RHD and the transmembrane hairpin in their structure but differ in their topologies indicating possible differences in their ER shaping functions [33,52,53,54] and possibly their localization [55,56]. REEP5, also known as DP1/Yop1p (Deleted in Polyposis1 or its yeast homolog Yop1) interacts with reticulons to form and stabilize ER membrane curvature by ‘wedging’ and oligomer formation [39]. REEP3/4 have been shown to be critical for ER tubulation during mitosis [52] apart from their role in ER clearing from metaphase chromatin [54]. REEP1 and REEP2 evidently are expressed specifically in neuronal or neuronal-like exocytotic tissue [57], and REEP1 interacts with Atlastin-1 and spastin proteins to form the ER network by both shaping ER tubules and mediating ER-microtubule (MT) interaction [51].

Atlastins constitute a family of dynamin related GTPases with structural similarity with mitofusins, another family of large GTPases. They have been reported to localize mainly on the ER membrane [58,59]. They mediate homotypic fusions of the ER membrane by forming three-way junctions and building the typical peripheral ER network [33,60,61,62,63]. They are, thus, significant for maintaining ER dynamics. There are three atlastin paralogs in mammals (ATL1, ATL2, and ATL3). Of these, ATL1, also known as the Hereditary Spastic Paraplegia (HSP) 3A (SPG3A) protein is abundantly expressed in cerebral cortex of brain and shows a general distribution in ER, while ATL2 and ATL3 mainly localize at three-way junctions [64]. The following mechanism of atlastin-mediated ER fusion has been proposed based on structure-function studies of Atlastin in Drosophila: Atlastins in two opposing membranes dimerize via their GTPases, which are bound to GDP [61,62,63]. The energy of hydrolysis post-GDP exchange powers conformational change in the atlastins. This increases the proximity between the two membranes, leading them to fuse as a result of membrane destabilization and curvature [33,62]. Atlastin1 has been shown to be a primary binding partner of Spastin, also known as SPG4, an ATPase [58,65], and along with the ER membrane resident REEP1, they mediate ER-MT interaction [51].

Spastin, encoded by *SPG4* or *SPAST* gene is a AAA ATPase (ATPase Associated with various Activities). Studies with HSP-associated mutations in the *SPG4* gene indicated Spastin’s role in regulation of MT dynamics via MT-severing [65,66,67,68,69]. Usage of alternative translation initiation sites can generate two isoforms of Spastin—a M1 isoform of 68 kDa or a 60 kDa M87 isoform [58,70]. Of these, the M1 Spastin, an integral membrane protein, harbors a hydrophobic domain that mediates its interaction with Atlastin-1 and REEP1. M1 expression is generally high in neurons in the brain, especially in spinal cord [70,71,72,73], and it has been found to localize to ER [53]. Apart from the wide range of cellular activities associated with AAA protein, an important function of Spastin is regulation of MT severing associated with formation, elongation, and maintenance of axons [65,74,75,76].

Cytoskeleton linking membrane protein, CLIMP63 (p63) is a 63 kDa membrane protein that mainly localizes at ER sheets. It acts as an intraluminal tether that holds the two ER membranes at a constant distance of ~30 nm in yeast and ~50 nm in mammalian cells [35,77,78,79]. CLIMP63 has a TM domain, an extended coiled coil domain and an N-terminal cytoplasmic segment. The sheet localization of the protein is possibly regulated by the luminal coiled-coil domain which also mediates oligomerization of CLIMP63. These oligomers possibly span across and determine the width of the luminal spacing of the sheets [35,77]. While CLIMP63 overexpression has been seen to cause a conspicuous increase in the number of ER sheets, its depletion reduces the width of ER sheet lumen to 25–30 nm without any significant effect on the ER sheet to tubule ratio [38]. It is important to emphasize the significance of combinatorial action of tubule inducing and sheet promoting proteins in maintaining the apt ratio of the two subdomains. Yeast deletion mutants of Rtn1 and Yop1 showed a massive expansion of sheets due to absence of curvature at the sheet edges [35,80]. Recently, using STED microscopy, CLIMP63 and reticulon 4A were shown to regulate the segregation of membrane-associated ER proteins from lumenal nanodomains along the peripheral ER tubules in human malignant epithelial and connective tissue cells [81].

True to its name, CLIMP63 mediates ER-MT interaction through its cytosolic domain [81,82,83]. In cultured hippocampal neurons, it has been shown to interact with MTs via MAP2 mediation in dendrites, hinting at a role in the variable distribution of ribosome-bound ER sheets between different regions of neurons [84]. While this implied a preferential assortment of ER sheets in dendrites, CLIMP63 knockdown resulted in an increase in ER tubules in axons, suggesting a role of CLIMP63 mediated ER-microtubule contacts in axonal distribution of ER tubules [85,86].

Several other proteins have been identified for their role in ER shaping and dynamics apart from those mentioned above. A few of these are Lunapark, p180, Kinectin [87], the RabGTPases, Rab10 and Rab18, FAM134B/RETREG1 [88], and Ataxin2 [89,90]. Lunapark (Lnp1) localizes to three-way junctions in the ER and stabilizes the junctions [91,92]. Rab10 has been shown to regulate ER dynamics with its role in ER tubule formation and fusion [93]. Disruption of Rab18 (activated by Rab3GAP complex for ER recruitment) functions disturbed the ER networks and altered the distribution of ER sheets [93,94]. It is important to consider here that even though functions of different proteins culminate in a similar ER morphology, for example, generation or fusion of ER tubules, or formation of ER sheets, the mechanism of action of these proteins might differ significantly from each other [35]. Initially identified as a ribosome receptor on the ER, p180 has been implicated in ER–MT interaction that regulates ER distribution [95,96]. In neurons, p180 has been shown to mediate MT stabilization and thus regulated ER distribution associated with axonal specification [85].

### 3.2. Sheet Stabilization by Ribosomes

Analysis of thin-section TEM images of different cell types show that intact sheets have the highest density of bound ribosomes compared to that on fenestrated sheets, which is more than the ribosome density on tubular membranes [27]. The localization of ribosomes and ribosome clusters also possibly affect the ER shape and dynamics. ER sheets were found to be disrupted upon release of the ER bound ribosomes upon treatment with puromycin, a potent translation inhibitor that targets the ribosomes [37]. Polyribosomes are also important for segregation of sheet-enriching proteins into ER-sheet cisternae [38]. It is also known that proteasomes compete for binding of the ER membrane with ribosomes [97]. This is mediated primarily by both, ribosomes and proteasomes, binding the Sec61 channel of the protein translocation machinery.

### 3.3. Role of Cytoskeleton in ER Dynamics

The cytoskeleton has an indispensable role in organization, distribution, and dynamics of the ER tubular network in cells in vivo [98,99]. MTs interact with ER to form new ER tubules and support ER dynamics, especially the peripheral tubular network [35,99,100]. Depolymerization of MTs result in loss of ER tubules, culminating in the ‘drawing in’ of the peripheral ER into perinuclear sheets [39,99] and increases ER sheet to tubule ratio [101].

ER–MT interaction can be in the form of (i) sliding, (ii) via a tip attachment complex (TAC), or (iii) through ring rearrangements. Sliding is a motor-driven movement of ER along MTs [102,103,104,105] and occurs preferentially over acetylated MTs [106]. Movement by TAC involves attachment of a tip of an ER tubule to the plus end of a MT and the ER tubule grows and shrinks along with the attached MT [104]. The TAC system includes a MT plus end-binding protein EB1, which connects with stroma interacting molecule 1 (STIM1), an integral ER membrane protein to mediate this movement [105,107,108]. Ring arrangements involve MT-dependent movement of ring structures of ER tubules. The rings are comprised of two three-way junctions. One of these is seemingly immobile, attached to the reticular ER network, while the second junction is dynamic and slides along the reticular ER towards the relatively fixed junction, sometimes resulting in ring closure [35,109]. These ER rings have important implications in ER contacts with mitochondria and endosomes. They are positioned around mitochondria or endosomes and generally result in fission of these organelles at the site of enclosure [35,110,111,112].

An opposite effect from MT depolymerization is however seen by actin depolymerization using the drug Latrunculin A, which showed a decreased sheet-to-tubule ratio, culminating in a more reticular network in a human hepatic cell line [101]. Dynamic actin arrays are controlled by Myosin 1c, and function in stabilizing ER sheets by keeping them immobile.

### 3.4. ER Contacts

The ER membrane has extensive regions of close appositions with multiple organelles forming heterotypic contact sites [113,114]. These sites provide an alternate means for faster inter-organelle contacts in cells, with a maximum distance of usually about 30 nm between the interacting organelles that are close enough, but not fusing [23,114,115,116]. A wide variety of functions have been attributed to ER contact sites including lipid exchange, Ca^2+^ signaling [23,35,86,117,118], autophagy regulation [119], and regulation of glucose homeostasis [120].

ER-mitochondria contacts (Mitochondria-ER contacts, MERC) or more commonly known Mitochondria associated membranes (MAM) were one of the earliest observed among ER contacts [121,122]. Many membrane proteins have been found to mediate or regulate the structure and function of ER-mitochondria contact sites [123]. Apart from the varied functions attributed to MAMs, such as an import of phospahtidylserine from the ER to mitochondria and regulation of calcium homeostasis, autophagy, and apoptosis, a recent study reports the presence of a microRNA profile specific to these contacts in human and rat brains with a change in this profile downstream of traumatic injury and cellular stress [124].

Lipid droplets (LD) serve as cellular storehouse for neutral lipids. Structurally, they consist of a hydrophobic core of these lipids surrounded by a phospholipid monolayer [86,125,126]. The lipids are synthesized within ER and in mammals, the LDs either bud off or remain in contact with the ER [127,128]. These contacts are mainly mediated by tubular ER [129,130] with studies implicating a role of membrane curvature in catalyzing LD assembly [131]. Seipin [131,132,133], Snx14 [134], DFCP1 [135], and Rab18 [136] are a few of the proteins associated with initiation and regulation of ER-LD contacts [86]. These are also highly expressed in brain [137,138,139,140]. ER-LD contacts have been shown to be highly stable [132] and, recently, tripartite contacts between ER, LDs, and early endosomes have been reported [141] in cultured mammalian cells.

ER-PM contacts are a ubiquitous entity seen in most cells including neurons [142]. Broadly referred to as ‘Cortical ER’, they are an abundant and characteristic subdomain of the yeast ER [142,143]. The cortical ER morphology is intermediate between that of sheets and tubules, characterized by regions of highly fenestrated sheets and regions of high curvature [35]. The close appositions between ER and PM had been noted and even speculated about long back [144,145], but the actual functions of these dynamic interactions could only be studied much later. The functions of such close ER-PM appositions include metabolic regulation and even cell migration [146].

## 4. ER Morphology in Neurons with a Focus on Mature Neurons

**Figure 3:**
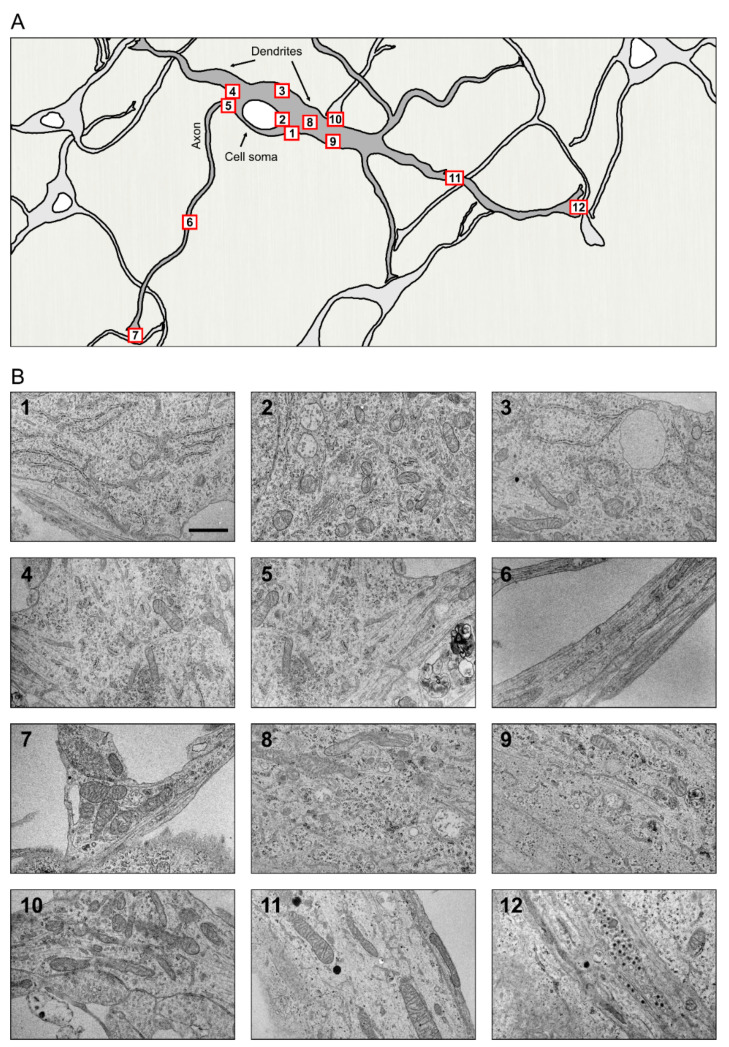
Neuronal ER comes with different shapes and interaction partners throughout the cell. (**A**) A schematic drawing of a neuron comprising soma, axon and dendrites. Numbered boxes in the drawing denote area, where the corresponding micrograph in (**B**) is taken. (**B**) Thin section transmission electron micrographs from various areas from a cultured primary midbrain dopaminergic neuron from postnatal mice expressing GFP under the TH promoter. The ER is visible in all compartments of the neuron. Note the differences in the ER sheets and tubules in the somatodendritic areas compared to various areas of the axon. For interactive image, go to https://www.helsinki.fi/en/researchgroups/organelle-structure/research#section-106929 (accessed on 19 April 2021) eMagnification bar, 200 nm.

The basic structure of a neuron is understood to comprise the neuronal cell body or soma and its two types of specialized extensions or neurites, the axon and dendrites with variations, such as in cell size, shape, and number of branches seen between neurons in different regions of the brain [147]. The morphology of any organelle can be understood to a great extent by looking at the shape and general condition of the cell that harbors it. Evidently, the organelle architecture has to: (i) comply with the spatial restrictions of the cell, (ii) facilitate optimal functioning of the organelle, and (iii) render flexibility to the organelle to expand its function as the situation demands. The neuronal ER is an excellent example of such a well-adapted adept organelle that is found distributed throughout this polarized cell, from the widest of regions in the cell body to its narrowest tubular axonal projections of constant width and in its profusely branched dendrites with tapering ends. The highly crowded cytoplasm of neurons exhibits some of the most specialized and dynamic structural arrangements of the ER (Figure 3).

### 4.1. ER in Neuronal Soma

ER in the neuronal cell body or soma appears as an interwoven, fused network of cisternae and tubules spread throughout the cytoplasm. Ribosome-studded ER sheets are predominant in the neuronal cell body [23,41,148] and these sheets are arranged roughly parallel to each other [148]. While the ER in periphery are seen as mostly sheets, the ER near the Golgi in the pericentriolar region are more tubular [41]. The highly packed arrangement of ER sheets in the soma (i) provides extensive area for ribosome attachment to facilitate protein synthesis, and (ii) offers a compact arrangement for the central protein synthetic machinery, which is possibly an adaptation to the available cytoplasmic volume of the cell body which is usually minimal compared to that of the neurites in total.

The ER in soma and proximal dendritic regions were first referred to as Nissl bodies (or tigroids) when observed under light microscope by Franz Alexander Nissl as basophilic granular masses in the neuroplasm [1,15]. The Nissl bodies, also referred to as Nissl substance, were observed to consist of stacks of ER sheets with rosettes of polysomes, which are either in close contact with the ER membrane or scattered in the intervening matrix between these stacks [15,149]. Electron microscopic examinations in different types of neurons have shown that ER profiles in the Nissl body consists of tubules, cisternae with occasional fenestrations, and vesicles weaved into an interconnected complex network. The 3D-EM studies have shown that at the periphery of these aggregates, the ER is mostly plate-like cisternae [150]. The adjacent rows of reticular sheets in these stacks are fused, and despite their curves and bends, they are maintained parallel to each other. At the center, the ER network is more complex with ‘ribbon’ and ‘thread’ like cisternae. Distribution of the Nissl bodies has been found to vary in its size, quantity, form, and degree of orientation of the ER in different types of neurons [15,151]. For example, the Nissl bodies in neurons of the sympathetic ganglion are usually smaller than those in the dorsal root ganglion cells and the motor neurons [15]. The ER display different types of orientation, exhibiting a highly layered arrangement or a randomly oriented reticula within a cell itself depending on its metabolic state or functional specialization [150].

The ER sheets with the associated polysomes serve as major sites of neuronal protein synthesis. Chromatolysis, the fission or fragmentation of the Nissl substance, including the ER sheets and the ribosomes, implies a disruption in the protein machinery along with other changes in the cell. It is a major event in neurons with axonal injury with the extent of chromatolysis being linked to severity of the neuron injury [152]. Identified mostly as a precursor of apoptosis, chromatolysis is also seen in neurons because of demyelination, cell toxicity, and infections [153]. It is seen in the motor neurons in Amyotrophic lateral sclerosis (ALS) patients [154], possibly as a culmination of aberrations in ER morphology starting with enlargement of the Nissl substance in early stages of sporadic ALS [155].

Another characteristic feature of the ER in neuronal soma is the extensive contacts between ER and the PM, mediated predominantly by large cisternae. These cortical ER or subsurface cisternae referring to the ER cisternae closely apposed with PM cover over 10% of the PM in cell body [23], but decrease reversibly following excitation [156]. The lumen of these PM-associated ER cisternae were found to be quite variable, ranging from 25nm to even narrower dimensions between the binding cisternal membranes, as seen at maximum possible FIB-SEM resolution [23]. An interesting feature observed by Wu et al. was the apposition of these thin ER with wide ER cisternae on the opposite side of the PM or their alignment with similar ‘thin’ ER in adjacent neurons.

### 4.2. ER in Axons

The distribution of ER subdomains between the somatodendritic and axonal regions perhaps exemplifies one of the finest segregation processes one can encounter in a cell. While the soma and dendrites abound in ribosome studded ER sheets, these large structures are mostly kept away from the narrow confinements of the axons. Ultrastructural studies have shown that the axon hillock is mostly devoid of ribosome studded ER though ribosomes are found scattered in this region [157,158]. Stacked ER sheets are found in the axon initial segments of some CNS neurons, such as the dentate granule cells and cortical principal cells [3,159,160]. Axonal ER is predominantly tubular, running parallel along the axonal length with intermittent small cisternae such as in the synaptic varicosities [23]. The larger axons have a network of interconnected tubules while a single axonal tubule is found in the thinner parts of the axon [23,161,162,163]. The ER tubules show extensive branching in the synaptic varicosities in the distal axon [23]. Using Automated Tape Collecting Ultramicrotomy (ATUM), a small but distinct population of very narrow ER tubules ranging between 20 and 30 nm in width were found in both central and peripheral system neurons [164]. These narrow tubules are particularly abundant in the axons, maintaining the ER density relatively constant along the axon length [23,163,164]. Regions of such ‘narrow constriction’ could have functional implications in the efficiency of transport of substances along the length of the tubule.

ER in the axons nonetheless makes contact with several membrane bound organelles. The tubular ER network is found to surround other organelles including mitochondria, synaptic vesicles, and other organelles at the nerve terminals [23]. Experiments with the fluorescent dye DiOC6 to study ER in growth cones of cultured neurons indicated the presence of highly dynamic tubular or tubulovesicular ER elements in close parallel alignment with the microtubules in periphery. This ER-MT association possibly contributes to transport of other organelles in the growth cone that culminates in axon elongation. The axon growth cones contain some dynamic network of ER tubules that move parallelly to the microtubule bundles during extension [165].

### 4.3. ER in Dendrites

Stemming from the Greek word ‘dendron’ meaning ‘tree’, the aptly named dendrites are highly branched post synaptic protoplasmic extensions of the neurons that connect and communicate with other neurons. Most of the neurons bear multiple dendritic projections and these have a distinct structural and biochemical identity. Formed after the development of an axon [166,167], the dendrites harbor their own distinct set of organelles and protein content. Compared to the axons, their shafts become thinner as their distance from the cell body increases [168]. While the axonal ER astonishes with its distinct narrow and tubular morphology that is in perfect sync with the structural and functional demands of the, at times, extremely long unwinding axons, the dendritic ER confounds with its complex arrangements that serve in ensuring a well-sorted dendritic cargo at the highly arborized dendritic extremities.

ER in the proximal somatodendritic regions is mostly ribosome-rich while the distal dendritic regions mostly contain ribosome free tubular ER with occasional cisternae [23]. In general, along the dendrite length, the ER presents an uneven terrain of tubules with distinct microdomains [169].

Dendritic spines are neuronal protrusions from the dendrites that serve as post synaptic sites of more than 90% of excitatory glutamatergic synapses in the mammalian brain [170]. Originally described by Cajal in 1888, dendritic spines have garnered much attention recently as they have been implicated in learning and memory owing to their structural plasticity [171]. Morphological changes are seen in dendrites and dendritic spines in hippocampus post traumatic brain injury and treatments [172]. The area occupied by dendritic ER corresponds to the number of spines or synapses in the particular dendritic segment [169]. Larger spines have the ER network reaching into their head region while the smaller ones have ER only until their neck, connected in both cases by a single tubule with rest of the ER network [23].

The spine apparatus found in mature dendritic spines of cortical and hippocampal neurons in the CNS [173] was first described by Gray in 1959 and initially suggested as a calcium sequestering organelle in the dendritic spines by Fifková [174,175]. It consists of stacks of ribosome free ER separated by dense-staining bars containing synaptopodin protein [176,177]. The spine apparatus is continuous with the ribosome free ER tubules of the dendritic trunk [178]. Serial electron microscopy and subsequent 3D analysis have shown that the spine apparatus is present in > 80% of the large spines in adult rat hippocampal neurons [179]. Regulation of calcium, synthesis of proteins, and posttranslational modification of proteins are some of the functions associated with the spine apparatus [177]. The spine apparatus is highly dynamic, as seen during activation of NMDA receptors in the hippocampal neurons, which causes functionally significant changes in the spine morphology and volume [180,181].

The ER near dendritic spines and branchpoints is another example of how organelle architecture is modulated to exert specific functions. In dendrites, the ER is spread as a dynamic network of anastomosing tubules extending to several hundreds of microns in the dendritic branches and spines [17,182]. Modulated through CLIMP63 phosphorylation, this complex ER architecture works together with the complicated dendritic geometry to act as a mesh that influences cargo mobility, local membrane composition, and dendritic branching [183].

Recently identified RAVs, the novel ER subdomain seen in different cell types, including primary cortical neurons and developing primary hippocampal neurons [31], were found to be ER derived, mobile vesicles associated with 80S mammalian ribosomes and connected to the main ER network through thin tubules or three-way junctions. In neurons, they were found in the cell periphery in dendrites. These translationally active ER structures are postulated to participate in local protein synthesis as part of the activity-dependent plasticity and modulation at the synapse [31,184,185]. Interestingly, these structures were not seen in the axons.

### 4.4. ER Contacts in Neurons

Though reported earlier, the first detailed account on structure and proposed functions of ER-PM contacts in neurons came from Rosenbluth [186] who called them ‘subsurface cisterns’ (SSC) because of their location and morphology. SSCs are membrane bound, mainly ribosome free flat structures found in proximity with the PM, and without any other organelles or ribosomes in the intervening space between them. The lumen of SSC is extremely narrow except at the swollen edges where its two surfaces are connected with each other, and where ribosomes are also seen attached to the membranes. The regions of such narrow lumen appear almost as regions of ER with non-existent lumen [23,187]. Barring the area between ER and PM, mitochondria and stacks of ER sheets are found in the vicinity of these contact sites. SSCs are connected to the rest of the ribosome studded ER in the cell and occur in all cells but were found to be particularly abundant in neurons. Interestingly, apart from the basic common features, the SSCs reportedly have some neuron type specific differences in their location. In spinal cord neurons, they were found to underlie synaptic boutons covering variable lengths of the boutons beneath the PM. The cholinergic C boutons connecting with spinal motor neurons are anatomically characterized by the presence of SSCs. The cisterns are associated with calcium homeostasis in the region and changes in the C bouton system, including its size, have been linked to motor neuron diseases such as ALS [188,189,190]. The Purkinje cell has an elaborate system of well-developed hypolemmal cisterna. SSC is considered as a part of the hypolemmal cisternae which encompasses the extensive network of ribosome devoid cisterna lying parallel to the PM at a distance of about 600nm below it [187]. As studies across different neuronal types indicate, ER-PM interactions are most abundant in cell soma and proximal regions of dendrites compared to those in the neurites [23,142]. Both tethering proteins and the proteins concerned with functions of these interactions are present at the contact sites. How differences and changes in ER morphology at these ER-PM contact sites in different neurons underlie degenerative changes need to be studied further.

In neurons, MAMs or MERCs are the most abundant among ER contacts. The ER is mainly ribosome free and tubular at these sites [23,191]. Thus, the most common aberrations in ER observed at ER-mitochondria contact sites are due to defects in tubule shaping proteins or factors. Some of the ER-mitochondrial tethering proteins are Mfn2, VAPB-PTPI51, and PDZ58 [86,123]. ER-MAM in neurons facilitate several processes like mitochondrial fission [110], calcium signaling, regulation of autophagy [192], neuronal homeostasis [193], and have also been speculated to contribute to neurotransmission [194]. MERCs have been seen to be altered in various neurodegenerative conditions [195] including Alzheimer’s disease [196].

The preferential movement of ER over acetylated MTs is interestingly of significance for establishment of polarity during early development in neurons in vitro. While the mature neurons have a widespread distribution of acetylated microtubules, the preferential localization of acetylated MTs to future axon projections also seem to lay the way for subsequent movement of ER tubules to axons [85,197]. The actin cytoskeleton also regulates ER organization, dynamics, and function [101,198], as seen in the case of the motor protein, Myosin-Va (Myosin5A), which has been shown to deliver ER as cargo into the spines in Purkinje neurons along actin filaments thereby aiding in the formation of spine apparatus [199]. Spine apparatus in dendrites are integral to synaptic plasticity, implying a requirement for transport of ER into the spines.

In neurons, LDs have been found under stressed conditions such as hypoxia, starvation, increased reactive oxygen species (ROS), or due to defects in lipid production machinery [200,201,202]. Apart from this, LDs have been readily visualized in glial cells in both central and peripheral systems [200,202]. Studies have shown that the astrocyte glial cells serve to defend the overstimulated or stressed neuron from impending fatty acid toxicity [203]. Under normal conditions, neurons are not efficient energy-storing cells and lack the lipid reserves found in other cell types, including glial cells. Periods of continuous stimulation triggers toxic responses culminating in build-up of fatty acids. This has been seen to induce LD formation in surrounding astrocytes to (i) take up the lipoprotein-FA lipoprotein secreted by the overstimulated neurons, and (ii) metabolize their LD reserves [200,202,203,204]. Nonetheless, there have been reports on LDs in Drosophila axons, rodent cortical neurons, and cortical neuron cultures [86]. Defects in lipid metabolizing and formation machinery, including Seipin have been implicated in motor neuron degenerative diseases [205,206,207]. Apart from these, mutations in the ER shaping proteins, Reep1, Atlastin 1 and Spastin [208,209,210] have been seen to cause defects in LD formation from ER. This could imply a disrupted ER-LD contact underlying degenerative diseases such as HSPs [86]. More studies on ER-LD in neurons need to be done to understand ER-LD interactions in other disorders including Parkinson’s Disease, in which differential expression of the tethering protein Seipin has been reported [211,212].

## 5. ER Heterogeneity between Neuronal Phenotypes

The folding demand and quantity of protein synthesis is drastically different in different cells in the human body. For example, in pancreatic β-cell granules insulin concentrations are very high, at about 40 mM [213,214]. In neuronal tissues none of the secreted proteins reach these concentrations. Neurons do not have such a high quantity demand for any protein to be synthesized and secreted. Neurons can be up to 1 m in length, and they can have thousands of synapses, and a single neuron can have a huge number of proteins. Therefore, protein folding quality demand is high to produce various kind of proteins such as, e.g., cytoskeleton proteins and complex PM receptors [215]. Indeed, neurons are extensively classified based on differences in their structure and function. Differences in different neuronal phenotypes are also observed in architecture and distribution of the protein synthetic machinery, the ER, and ribosomes. There are important basic questions for future studies that need to be answered. How do the structural variations translate to functional differences? Does a specific type of ER arrangement make a specific neuronal phenotype more susceptible to degeneration?

Some studies have already been conducted. Apart from the above-mentioned variations in ER organization in different neuronal phenotypes, comparative electron microscopy study of dopaminergic and non-dopaminergic neurons in the substantia nigra showed a relatively dense distribution of stacked ribosome studded ER and abundant free ribosomes in the dopamine neurons. The non-dopamine neurons appeared lighter with a lesser amount of ‘rough’ ER or free ribosomes clustering together [18]. Could this be related to substantia nigra dopaminergic neurons being more vulnerable to neurodegeneration?

Although the general composition of the ER is quite well known, little is known about differences in most ER proteins and lipids between cell types, and particularly between neuronal phenotypes and subpopulations. As quantitative methods are becoming more sensitive, large-scale single-cell proteomics and lipidomics will be paramount for uncovering all main components of the ER and their ratios [216]. Comparative studies will yield insights into the structural and functional differences of the ER of neuron and glial cell subtypes which may contribute to our understanding of cellular vulnerability in neurodegenerative disease. Rodent primary neurons and induced pluripotent stem cell-derived neurons will be a good starting point, as the isolation of neurons with all of its axons and dendrites from brain tissue is very challenging.

## 6. ER Morphology and Neurodegeneration

Disruption of the ER function and chronic ER stress are suggested to underlie progression of many human diseases. In neurodegeneration, many of the disease associated alterations in the neuronal proteostasis can be due to changes in the ER and dysfunction in Ca^2+^ homeostasis. Neurodegenerative diseases are often also referred to as protein misfolding disorders due to the common pathology of accumulation of misfolded proteins. Nevertheless, aging has been associated with an increase in the accumulation of misfolded proteins in the ER lumen due to the reduction in the buffering capacity of the proteostasis mechanisms [217]. Decreased protein folding capacity in aging can increase the risk of neurodegeneration. There are studies that suggest that aggregates of alpha-synuclein co-localize with ER luminal proteins, e.g., GRP78 [218,219], however more studies are needed in a physiologically relevant manner to conclude whether normally cytosolic protein aggregates accumulate into the ER lumen. The accumulation of the misfolded proteins leads to generation of ER stress and activation of the unfolded protein response (UPR), which has been linked to the pathophysiology of protein misfolding disorders [220]. ER stress leads to expansion of the ER membrane to withstand the accumulation of proteins. Cellular stress and pathological conditions may also cause ER phagy related ER whorls or tubular ER aggregation, independent of ER stress [221,222,223]. Other morphological changes of the ER have also been reported in response to various stressors causing neurodegeneration. There are multiple studies linking altered ER structure to Alzheimer’s disease, amyotrophic lateral sclerosis (ALS), Huntington’s disease, Parkinson’s disease, and HSPs [191]. As examples, dysfunctional tubular ER has been observed in Alzheimer’s disease [224], enlargement of the ER in Huntington’s disease [225] and an ER-mitochondria tethering protein, which also regulates tubular ER morphogenesis and dynamics is linked to both ALS and Parkinson’s disease [226,227]. HSPs, as previously mentioned, are caused by mutations in proteins which directly regulate ER morphology. In fact, there seems to be a multitude of associations between proteins regulating ER morphology, ER calcium and MERCs in many neurodegenerative diseases, particularly HSPs and Parkinson’s disease, which are discussed further. Nonetheless, there is an unmet need to study the relationship of ER morphology to neurodegeneration in more detail.

### 6.1. Hereditary Spastic Paraplegias

HSPs is a large group of inherited diseases that are generally characterized by the degeneration of the longer upper motor neurons leading to lower limb weakness and spasticity [228,229]. HSP is a highly genetically heterogeneous disease, but a majority of the cases are caused by mutations in proteins that take part in the organization and generation of tubular ER [86,230]. For example, mutations in the ER-shaping proteins REEP1, Reticulon 2 (RTN2), spastin, and Atlastin 1 (ATL1) lead to different HSP subtypes. The most common mutations in HSP are in spastin (SPG), atlastin-1 (ATL-1), and REEP1. Mutations in the *SPG4* gene that causes a defect in the ATPase activity leads to aberrations in microtubule arrangement and affects ER-MT interaction culminating in alterations in ER distribution [51]. Some of the ER-shaping proteins can be found on MAMs and so mutations in them affect the formation and function of the contact sites [55]. In all, the mutation landscape that on a large scale is linked to defects in the function and development of ER indicates ER having a central role in the HSP pathology.

Mutation of atlastin-1 (ATL1) protein leads to SPG3A, which is the second most common subtype of HSP. Studies done on Drosophila motor neurons have showed that mutations and abnormal expression of Atlastin leads to dysfunction of the release and storage of synaptic vesicle and change in the morphology of axonal ER networks [231,232]. In knock out models of REEP1, the motor neurons had a reduced complexity and organization of ER [233]. REEP1 has also been detected at the MAMs, which may indicate that its mutation might also affect the Ca^2+^ and lipid exchange between ER and mitochondria [55].

### 6.2. Parkinson’s Disease

Parkinson’s disease is a chronic progressive neuron disease, and the second most common neurodegenerative disease. It is a heterogeneous disorder with widespread pathology, and both ER and the mitochondria have been linked to the pathogenesis [234]. UPR activation markers have shown to be increased in neurons of Parkinson’s disease patients [235]. The markers were colonized with diffuse alpha-synuclein staining, which are aggregated and found in Lewy bodies and neurites in Parkinson’s disease. Lewy body pathology is common in Parkinson’s disease and parts of the ER, or omegasomes, have been found in Lewy bodies [236]. Parkinson’s disease can be modeled by toxins and genetic models [237], and UPR has also been shown to be activated in cellular models of Parkinson’s disease [218,219,238]. These findings can suggest that UPR activation is an early event in neurodegeneration [235], but this needs more mechanistic studies.

A neuropathological characteristic associated with the disease is the progressive loss of dopaminergic neurons in the pars compacta of the substantia nigra, which is the leading cause of the movement disorder aspect. The reason behind the selective death of substantia nigra pars compacta dopaminergic neurons in Parkinson’s disease has eluded scientists for years. Several studies indicate a role of Ca^2+^ signaling in Parkinson’s disease pathogenesis and some also link it to the selective neuron vulnerability [239,240]. The lacking intrinsic Ca^2+^ buffering capacity of these neurons makes them vulnerable to high Ca^2+^ levels and indicate that they might also be sensitive to ER stress [241]. ER and MAMs are important in controlling the intracellular Ca^2+^ homeostasis. Alpha-synuclein has been shown to disrupt the connection between the mitochondria and ER by binding to VAPB, which is a protein that, together with PTPIP51, acts as scaffolds that link the organelles [227]. An overexpression of familial and wild-type PD mutant alpha-synuclein disrupts the VAPB-PTPIP51 connections between the organelles, which loosens ER-mitochondria connections. This leads to a decreased level of Ca^2+^ exchange and ATP production in the neurons, which can be fatal for the sensitive substantia nigra dopaminergic neurons.

Mutations in *SNCA*, the gene that produces alpha-synuclein, was the first mutation linked to PD and has also been linked to the ER’s effect on the disease [242]. Studies have shown that overexpression of alpha-synuclein inhibits COPII vesicle trafficking of proteins between ER and Golgi apparatus [243,244]. This has also been shown to modulate UPR, by inhibiting ATF6 production, as it uses COPII for the ER-Golgi transportation. Mutations of *MCTP2* gene is a risk factor for early onset PD development. Its potential role in tubular ER formation indicates a possible pathological role of disrupted ER organization in dopaminergic axons [191].

In the study by Domesick et al., [18] discussing the possible significance of differences in ER arrangement in the dopaminergic and non-dopaminergic neurons, the authors suggest investigating the morphological basis of the functional and metabolically distinguished subcategories among different dopaminergic neurons. It would be interesting to further explore differences in ER morphology between the DA neurons from different regions in SN and VTA to understand their selective susceptibility to degeneration in PD.

## 7. Concluding Remarks

The ER with its different structural functionally significant domains is integral to the survival of a cell. Many membrane proteins are important for shaping these domains [40,245,246]. There is now ample evidence that changes in neuronal ER morphology affect many different functions of the ER which have been linked to neurodegeneration. From the information available up to now, the most evident morphological alterations hinted are in the case of ER contacts, especially of ER-mitochondria contacts [86,247] wherein the ER at the contact sites is affected leading to aberrations in the functions that these contacts normally facilitate. Barring the basic structural similarity, the type and abundance of these contact sites are expected and, in some cases, already shown to be variable even in cells of the same type under different conditions. It would thus be an interesting approach to study the differences in region specific ER contact sites in different neuronal subtypes to understand if such variations underlie the differential susceptibility of specific neurons to degeneration, i.e., make some types of neurons more vulnerable to degenerative changes. Here, we propose a systematic classification of the neurons based on their ER morphology including organization and ER-organelle contacts, which could serve as a useful resource to trace their varied susceptibility to neurodegenerative changes.

This is by no means an exhaustive resource of information hitherto available on ER morphology and dynamics. Several excellent reviews have been published recently on ER structure and function in normal and aberrant conditions in neurons and neurodegenerative diseases, including comprehensive tables listing the tubular ER-related proteins, their localizations and contacts [86,191,216]. Currently available data linking neurodegenerative diseases with an underlying ER aberration suggest a prominent role of defective ER shaping proteins. The actual consequence of these defects on the ER morphology needs to be explored further. Presently available as well as future innovations in imaging techniques and data analysis solutions have a significant role in such studies.

## References

[1] Bhattacharyya K. (2012). Eminent Neuroscientists: Their Lives and Works.

[2] Sotelo C. (2003). Viewing the brain through the master hand of Ramon y Cajal. Nat. Rev. Neurosci..

[3] Berridge M.J. (1998). Neuronal calcium signaling. Neuron.

[4] Knoll M., Ruska E. (1932). Das Elektronenmikroskop. Z. Für Phys..

[5] Veratti E. (1961). Investigations on the fine structure of striated muscle fiber read before the Reale Istituto Lombardo, 13 March 1902. J. Biophys. Biochem. Cytol..

[6] Porter K.R., Claude A., Fullam E.F. (1945). A study of tissue culture cells by electron microscopy: Methods and preliminary observations. J. Exp. Med..

[7] Palade G.E. (1952). The fine structure of mitochondria. Anat. Rec..

[8] Porter K.R. (1953). Observations on a submicroscopic basophilic component of cytoplasm. J. Exp. Med..

[9] Shibata Y., Voeltz G.K., Rapoport T.A. (2006). Rough sheets and smooth tubules. Cell.

[10] Griffiths G. (1993). Fine Structure Immunocytochemistry.

[11] Copeland D.E., Dalton A.J. (1959). An association between mitochondria and the endoplasmic reticulum in cells of the pseudobranch gland of a teleost. J. Biophys. Biochem. Cytol..

[12] Kitai S.T., Kocsis J.D., Preston R.J., Sugimori M. (1976). Monosynaptic inputs to caudate neurons identified by intracellular injection of horseradish peroxidase. Brain Res..

[13] Nauta W.J., Gygax P.A. (1954). Silver impregnation of degenerating axons in the central nervous system: A modified technic. Stain Technol..

[14] Peters A. (2007). Golgi, Cajal, and the fine structure of the nervous system. Brain Res. Rev..

[15] Palay S.L., Palade G.E. (1955). The fine structure of neurons. J. Biophys. Biochem. Cytol..

[16] Tsukita S., Ishikawa H. (1976). Three-dimensional distribution of smooth endoplasmic reticulum in myelinated axons. J. Electron. Microsc..

[17] Terasaki M., Slater N.T., Fein A., Schmidek A., Reese T.S. (1994). Continuous network of endoplasmic reticulum in cerebellar Purkinje neurons. Proc. Natl. Acad. Sci. USA.

[18] Domesick V.B., Stinus L., Paskevich P.A. (1983). The cytology of dopaminergic and nondopaminergic neurons in the substantia nigra and ventral tegmental area of the rat: A light- and electron-microscopic study. Neuroscience.

[19] Mrak R.E. (2002). The Big Eye in the 21st century: The role of electron microscopy in modern diagnostic neuropathology. J. Neuropathol. Exp. Neurol..

[20] Walker C.K., Roche J.K., Sinha V., Roberts R.C. (2018). Substantia nigra ultrastructural pathology in schizophrenia. Schizophr. Res..

[21] Abdollahzadeh A., Belevich I., Jokitalo E., Tohka J., Sierra A. (2019). Automated 3D Axonal Morphometry of White Matter. Sci. Rep..

[22] Salo R.A., Belevich I., Manninen E., Jokitalo E., Grohn O., Sierra A. (2018). Quantification of anisotropy and orientation in 3D electron microscopy and diffusion tensor imaging in injured rat brain. Neuroimage.

[23] Wu Y., Whiteus C., Xu C.S., Hayworth K.J., Weinberg R.J., Hess H.F., De Camilli P. (2017). Contacts between the endoplasmic reticulum and other membranes in neurons. Proc. Natl. Acad. Sci. USA.

[24] Zheng Z., Lauritzen J.S., Perlman E., Robinson C.G., Nichols M., Milkie D., Torrens O., Price J., Fisher C.B., Sharifi N. (2018). A Complete Electron Microscopy Volume of the Brain of Adult Drosophila melanogaster. Cell.

[25] Nakadate K., Tanaka-Nakadate S. (2015). Three-Dimensional Electron Microscopy Reconstruction of Degenerative Dopaminergic Neurons Surrounded by Activated Microglia in Substantia Nigra. Ultrastruct. Pathol..

[26] Voeltz G.K., Rolls M.M., Rapoport T.A. (2002). Structural organization of the endoplasmic reticulum. EMBO Rep..

[27] Puhka M., Joensuu M., Vihinen H., Belevich I., Jokitalo E. (2012). Progressive sheet-to-tubule transformation is a general mechanism for endoplasmic reticulum partitioning in dividing mammalian cells. Mol. Biol. Cell.

[28] Palade G. (1975). Intracellular aspects of the process of protein synthesis. Science.

[29] Lavieu G., Orci L., Shi L., Geiling M., Ravazzola M., Wieland F., Cosson P., Rothman J.E. (2010). Induction of cortical endoplasmic reticulum by dimerization of a coatomer-binding peptide anchored to endoplasmic reticulum membranes. Proc. Natl. Acad. Sci. USA.

[30] Terasaki M., Shemesh T., Kasthuri N., Klemm R.W., Schalek R., Hayworth K.J., Hand A.R., Yankova M., Huber G., Lichtman J.W. (2013). Stacked endoplasmic reticulum sheets are connected by helicoidal membrane motifs. Cell.

[31] Carter S.D., Hampton C.M., Langlois R., Melero R., Farino Z.J., Calderon M.J., Li W., Wallace C.T., Tran N.H., Grassucci R.A. (2020). Ribosome-associated vesicles: A dynamic subcompartment of the endoplasmic reticulum in secretory cells. Sci. Adv..

[32] Fiori M.G., Mugnaini E. (1981). Subsurface and cytoplasmic cisterns associated with mitochondria in pyramidal neurons of the rat dorsal cochlear nucleus. Neuroscience.

[33] Schwarz D.S., Blower M.D. (2016). The endoplasmic reticulum: Structure, function and response to cellular signaling. Cell. Mol. Life Sci..

[34] Shemesh T., Klemm R.W., Romano F.B., Wang S., Vaughan J., Zhuang X., Tukachinsky H., Kozlov M.M., Rapoport T.A. (2014). A model for the generation and interconversion of ER morphologies. Proc. Natl. Acad. Sci. USA.

[35] Westrate L.M., Lee J.E., Prinz W.A., Voeltz G.K. (2015). Form follows function: The importance of endoplasmic reticulum shape. Annu. Rev. Biochem..

[36] Phuyal S., Baschieri F. (2020). Endomembranes: Unsung Heroes of Mechanobiology?. Front. Bioeng. BioTechnol..

[37] Puhka M., Vihinen H., Joensuu M., Jokitalo E. (2007). Endoplasmic reticulum remains continuous and undergoes sheet-to-tubule transformation during cell division in mammalian cells. J. Cell Biol..

[38] Shibata Y., Shemesh T., Prinz W.A., Palazzo A.F., Kozlov M.M., Rapoport T.A. (2010). Mechanisms determining the morphology of the peripheral ER. Cell.

[39] Shibata Y., Voss C., Rist J.M., Hu J., Rapoport T.A., Prinz W.A., Voeltz G.K. (2008). The reticulon and DP1/Yop1p proteins form immobile oligomers in the tubular endoplasmic reticulum. J. Biol. Chem..

[40] Voeltz G.K., Prinz W.A., Shibata Y., Rist J.M., Rapoport T.A. (2006). A class of membrane proteins shaping the tubular endoplasmic reticulum. Cell.

[41] Rämö O., Kumar D., Gucciardo E., Joensuu M., Saarekas M., Vihinen H., Belevich I., Smolander O.P., Qian K., Auvinen P. (2016). NOGO-A/RTN4A and NOGO-B/RTN4B are simultaneously expressed in epithelial, fibroblast and neuronal cells and maintain ER morphology. Sci. Rep..

[42] Zhao J., Hu J. (2020). Self-Association of Purified Reconstituted ER Luminal Spacer Climp63. Front. Cell Dev. Biol..

[43] Oertle T., Huber C., van der Putten H., Schwab M.E. (2003). Genomic structure and functional characterisation of the promoters of human and mouse nogo/rtn4. J. Mol. Biol..

[44] Yang Y.S., Strittmatter S.M. (2007). The reticulons: A family of proteins with diverse functions. Genome Biol..

[45] Zurek N., Sparks L., Voeltz G. (2011). Reticulon short hairpin transmembrane domains are used to shape ER tubules. Traffic.

[46] Grumati P., Morozzi G., Holper S., Mari M., Harwardt M.I., Yan R., Muller S., Reggiori F., Heilemann M., Dikic I. (2017). Full length RTN3 regulates turnover of tubular endoplasmic reticulum via selective autophagy. eLife.

[47] Khaminets A., Heinrich T., Mari M., Grumati P., Huebner A.K., Akutsu M., Liebmann L., Stolz A., Nietzsche S., Koch N. (2015). Regulation of endoplasmic reticulum turnover by selective autophagy. Nature.

[48] D’Eletto M., Risuglia A., Oliverio S., Mehdawy B., Nardacci R., Bordi M., Di Sano F. (2019). Modulation of autophagy by RTN-1C: Role in autophagosome biogenesis. Cell Death Dis..

[49] Espadas J., Pendin D., Bocanegra R., Escalada A., Misticoni G., Trevisan T., Velasco Del Olmo A., Montagna A., Bova S., Ibarra B. (2019). Dynamic constriction and fission of endoplasmic reticulum membranes by reticulon. Nat. Commun..

[50] Saito H., Kubota M., Roberts R.W., Chi Q., Matsunami H. (2004). RTP family members induce functional expression of mammalian odorant receptors. Cell.

[51] Park S.H., Zhu P.P., Parker R.L., Blackstone C. (2010). Hereditary spastic paraplegia proteins REEP1, spastin, and atlastin-1 coordinate microtubule interactions with the tubular ER network. J. Clin. Investig..

[52] Kumar D., Golchoubian B., Belevich I., Jokitalo E., Schlaitz A.L. (2019). REEP3 and REEP4 determine the tubular morphology of the endoplasmic reticulum during mitosis. Mol. Biol. Cell.

[53] Park S.H., Blackstone C. (2010). Further assembly required: Construction and dynamics of the endoplasmic reticulum network. EMBO Rep..

[54] Schlaitz A.L., Thompson J., Wong C.C., Yates J.R., Heald R. (2013). REEP3/4 ensure endoplasmic reticulum clearance from metaphase chromatin and proper nuclear envelope architecture. Dev. Cell.

[55] Lim Y., Cho I.T., Schoel L.J., Cho G., Golden J.A. (2015). Hereditary spastic paraplegia-linked REEP1 modulates endoplasmic reticulum/mitochondria contacts. Ann. Neurol..

[56] Züchner S., Wang G., Tran-Viet K.N., Nance M.A., Gaskell P.C., Vance J.M., Ashley-Koch A.E., Pericak-Vance M.A. (2006). Mutations in the novel mitochondrial protein REEP1 cause hereditary spastic paraplegia type 31. Am. J. Hum. Genet..

[57] Hurt C.M., Björk S., Ho V.K., Gilsbach R., Hein L., Angelotti T. (2014). REEP1 and REEP2 proteins are preferentially expressed in neuronal and neuronal-like exocytotic tissues. Brain Res..

[58] Sanderson C.M., Connell J.W., Edwards T.L., Bright N.A., Duley S., Thompson A., Luzio J.P., Reid E. (2006). Spastin and atlastin, two proteins mutated in autosomal-dominant hereditary spastic paraplegia, are binding partners. Hum. Mol. Genet..

[59] Zhu P.P., Patterson A., Lavoie B., Stadler J., Shoeb M., Patel R., Blackstone C. (2003). Cellular localization, oligomerization, and membrane association of the hereditary spastic paraplegia 3A (SPG3A) protein atlastin. J. Biol. Chem..

[60] Wang S., Tukachinsky H., Romano F.B., Rapoport T.A. (2016). Cooperation of the ER-shaping proteins atlastin, lunapark, and reticulons to generate a tubular membrane network. eLife.

[61] Betancourt-Solis M.A., Desai T., McNew J.A. (2018). The atlastin membrane anchor forms an intramembrane hairpin that does not span the phospholipid bilayer. J. Biol. Chem..

[62] Bian X., Klemm R.W., Liu T.Y., Zhang M., Sun S., Sui X., Liu X., Rapoport T.A., Hu J. (2011). Structures of the atlastin GTPase provide insight into homotypic fusion of endoplasmic reticulum membranes. Proc. Natl. Acad. Sci. USA.

[63] Orso G., Pendin D., Liu S., Tosetto J., Moss T.J., Faust J.E., Micaroni M., Egorova A., Martinuzzi A., McNew J.A. (2009). Homotypic fusion of ER membranes requires the dynamin-like GTPase atlastin. Nature.

[64] Behrendt L., Kurth I., Kaether C. (2019). A disease causing ATLASTIN 3 mutation affects multiple endoplasmic reticulum-related pathways. Cell. Mol. Life Sci..

[65] Evans K., Keller C., Pavur K., Glasgow K., Conn B., Lauring B. (2006). Interaction of two hereditary spastic paraplegia gene products, spastin and atlastin, suggests a common pathway for axonal maintenance. Proc. Natl. Acad. Sci. USA.

[66] Errico A., Ballabio A., Rugarli E.I. (2002). Spastin, the protein mutated in autosomal dominant hereditary spastic paraplegia, is involved in microtubule dynamics. Hum. Mol. Genet..

[67] Evans K.J., Gomes E.R., Reisenweber S.M., Gundersen G.G., Lauring B.P. (2005). Linking axonal degeneration to microtubule remodeling by Spastin-mediated microtubule severing. J. Cell Biol..

[68] Hazan J., Fonknechten N., Mavel D., Paternotte C., Samson D., Artiguenave F., Davoine C.S., Cruaud C., Dürr A., Wincker P. (1999). Spastin, a new AAA protein, is altered in the most frequent form of autosomal dominant spastic paraplegia. Nat. Genet..

[69] Roll-Mecak A., Vale R.D. (2005). The Drosophila homologue of the hereditary spastic paraplegia protein, spastin, severs and disassembles microtubules. Curr. Biol..

[70] Claudiani P., Riano E., Errico A., Andolfi G., Rugarli E.I. (2005). Spastin subcellular localization is regulated through usage of different translation start sites and active export from the nucleus. Exp. Cell Res..

[71] Charvin D., Cifuentes-Diaz C., Fonknechten N., Joshi V., Hazan J., Melki J., Betuing S. (2003). Mutations of SPG4 are responsible for a loss of function of spastin, an abundant neuronal protein localized in the nucleus. Hum. Mol. Genet..

[72] Solowska J.M., Morfini G., Falnikar A., Himes B.T., Brady S.T., Huang D., Baas P.W. (2008). Quantitative and functional analyses of spastin in the nervous system: Implications for hereditary spastic paraplegia. J. Neurosci..

[73] Wharton S.B., McDermott C.J., Grierson A.J., Wood J.D., Gelsthorpe C., Ince P.G., Shaw P.J. (2003). The cellular and molecular pathology of the motor system in hereditary spastic paraparesis due to mutation of the spastin gene. J. Neuropathol. Exp. Neurol..

[74] Brill M.S., Kleele T., Ruschkies L., Wang M., Marahori N.A., Reuter M.S., Hausrat T.J., Weigand E., Fisher M., Ahles A. (2016). Branch-Specific Microtubule Destabilization Mediates Axon Branch Loss during Neuromuscular Synapse Elimination. Neuron.

[75] Riano E., Martignoni M., Mancuso G., Cartelli D., Crippa F., Toldo I., Siciliano G., Di Bella D., Taroni F., Bassi M.T. (2009). Pleiotropic effects of spastin on neurite growth depending on expression levels. J. Neurochem..

[76] Yu W., Qiang L., Solowska J.M., Karabay A., Korulu S., Baas P.W. (2008). The microtubule-severing proteins spastin and katanin participate differently in the formation of axonal branches. Mol. Biol. Cell.

[77] Klopfenstein D.R., Klumperman J., Lustig A., Kammerer R.A., Oorschot V., Hauri H.P. (2001). Subdomain-specific localization of CLIMP-63 (p63) in the endoplasmic reticulum is mediated by its luminal alpha-helical segment. J. Cell Biol..

[78] Schweizer A., Ericsson M., Bächi T., Griffiths G., Hauri H.P. (1993). Characterization of a novel 63 kDa membrane protein. Implications for the organization of the ER-to-Golgi pathway. J. Cell Sci..

[79] Schweizer A., Rohrer J., Slot J.W., Geuze H.J., Kornfeld S. (1995). Reassessment of the subcellular localization of p63. J. Cell Sci..

[80] West M., Zurek N., Hoenger A., Voeltz G.K. (2011). A 3D analysis of yeast ER structure reveals how ER domains are organized by membrane curvature. J. Cell Biol..

[81] Gao G., Zhu C., Liu E., Nabi I.R. (2019). Reticulon and CLIMP-63 regulate nanodomain organization of peripheral ER tubules. PLoS Biol..

[82] Klopfenstein D.R., Kappeler F., Hauri H.P. (1998). A novel direct interaction of endoplasmic reticulum with microtubules. EMBO J..

[83] Nikonov A.V., Hauri H.P., Lauring B., Kreibich G. (2007). Climp-63-mediated binding of microtubules to the ER affects the lateral mobility of translocon complexes. J. Cell Sci..

[84] Farah C.A., Liazoghli D., Perreault S., Desjardins M., Guimont A., Anton A., Lauzon M., Kreibich G., Paiement J., Leclerc N. (2005). Interaction of microtubule-associated protein-2 and p63: A new link between microtubules and rough endoplasmic reticulum membranes in neurons. J. Biol. Chem..

[85] Farías G.G., Fréal A., Tortosa E., Stucchi R., Pan X., Portegies S., Will L., Altelaar M., Hoogenraad C.C. (2019). Feedback-Driven Mechanisms between Microtubules and the Endoplasmic Reticulum Instruct Neuronal Polarity. Neuron.

[86] Fowler P.C., Garcia-Pardo M.E., Simpson J.C., O’Sullivan N.C. (2019). NeurodegenERation: The Central Role for ER Contacts in Neuronal Function and Axonopathy, Lessons From Hereditary Spastic Paraplegias and Related Diseases. Front. Neurosci..

[87] Santama N., Er C.P., Ong L.L., Yu H. (2004). Distribution and functions of kinectin isoforms. J. Cell Sci..

[88] Bhaskara R.M., Grumati P., Garcia-Pardo J., Kalayil S., Covarrubias-Pinto A., Chen W., Kudryashev M., Dikic I., Hummer G. (2019). Curvature induction and membrane remodeling by FAM134B reticulon homology domain assist selective ER-phagy. Nat. Commun..

[89] Del Castillo U., Gnazzo M.M., Sorensen Turpin C.G., Nguyen K.C.Q., Semaya E., Lam Y., de Cruz M.A., Bembenek J.N., Hall D.H., Riggs B. (2019). Conserved role for Ataxin-2 in mediating endoplasmic reticulum dynamics. Traffic.

[90] van de Loo S., Eich F., Nonis D., Auburger G., Nowock J. (2009). Ataxin-2 associates with rough endoplasmic reticulum. Exp. Neurol..

[91] Chen S., Desai T., McNew J.A., Gerard P., Novick P.J., Ferro-Novick S. (2015). Lunapark stabilizes nascent three-way junctions in the endoplasmic reticulum. Proc. Natl. Acad. Sci. USA.

[92] Chen S., Novick P., Ferro-Novick S. (2012). ER network formation requires a balance of the dynamin-like GTPase Sey1p and the Lunapark family member Lnp1p. Nat. Cell Biol..

[93] English A.R., Voeltz G.K. (2013). Rab10 GTPase regulates ER dynamics and morphology. Nat. Cell Biol..

[94] Gerondopoulos A., Bastos R.N., Yoshimura S., Anderson R., Carpanini S., Aligianis I., Handley M.T., Barr F.A. (2014). Rab18 and a Rab18 GEF complex are required for normal ER structure. J. Cell Biol..

[95] Ogawa-Goto K., Tanaka K., Ueno T., Tanaka K., Kurata T., Sata T., Irie S. (2007). p180 is involved in the interaction between the endoplasmic reticulum and microtubules through a novel microtubule-binding and bundling domain. Mol. Biol. Cell.

[96] Savitz A.J., Meyer D.I. (1990). Identification of a ribosome receptor in the rough endoplasmic reticulum. Nature.

[97] Kalies K.U., Allan S., Sergeyenko T., Kroger H., Romisch K. (2005). The protein translocation channel binds proteasomes to the endoplasmic reticulum membrane. EMBO J..

[98] Schroeder L.K., Barentine A.E.S., Merta H., Schweighofer S., Zhang Y., Baddeley D., Bewersdorf J., Bahmanyar S. (2019). Dynamic nanoscale morphology of the ER surveyed by STED microscopy. J. Cell Biol..

[99] Terasaki M., Chen L.B., Fujiwara K. (1986). Microtubules and the endoplasmic reticulum are highly interdependent structures. J. Cell Biol..

[100] Terasaki M., Song J., Wong J.R., Weiss M.J., Chen L.B. (1984). Localization of endoplasmic reticulum in living and glutaraldehyde-fixed cells with fluorescent dyes. Cell.

[101] Joensuu M., Belevich I., Rämö O., Nevzorov I., Vihinen H., Puhka M., Witkos T.M., Lowe M., Vartiainen M.K., Jokitalo E. (2014). ER sheet persistence is coupled to myosin 1c-regulated dynamic actin filament arrays. Mol. Biol. Cell.

[102] Allan V., Vale R. (1994). Movement of membrane tubules along microtubules in vitro: Evidence for specialised sites of motor attachment. J. Cell Sci..

[103] Vale R.D., Hotani H. (1988). Formation of membrane networks in vitro by kinesin-driven microtubule movement. J. Cell Biol..

[104] Waterman-Storer C.M., Gregory J., Parsons S.F., Salmon E.D. (1995). Membrane/microtubule tip attachment complexes (TACs) allow the assembly dynamics of plus ends to push and pull membranes into tubulovesicular networks in interphase Xenopus egg extracts. J. Cell Biol..

[105] Waterman-Storer C.M., Salmon E.D. (1998). Endoplasmic reticulum membrane tubules are distributed by microtubules in living cells using three distinct mechanisms. Curr. Biol..

[106] Friedman J.R., Webster B.M., Mastronarde D.N., Verhey K.J., Voeltz G.K. (2010). ER sliding dynamics and ER-mitochondrial contacts occur on acetylated microtubules. J. Cell Biol..

[107] Bola B., Allan V. (2009). How and why does the endoplasmic reticulum move?. Biochem. Soc. Trans..

[108] Grigoriev I., Gouveia S.M., van der Vaart B., Demmers J., Smyth J.T., Honnappa S., Splinter D., Steinmetz M.O., Putney J.W., Hoogenraad C.C. (2008). STIM1 is a MT-plus-end-tracking protein involved in remodeling of the ER. Curr. Biol..

[109] Lee C., Chen L.B. (1988). Dynamic behavior of endoplasmic reticulum in living cells. Cell.

[110] Friedman J.R., Lackner L.L., West M., DiBenedetto J.R., Nunnari J., Voeltz G.K. (2011). ER tubules mark sites of mitochondrial division. Science.

[111] Lee H., Yoon Y. (2014). Mitochondrial fission: Regulation and ER connection. Mol. Cells.

[112] Rowland A.A., Chitwood P.J., Phillips M.J., Voeltz G.K. (2014). ER contact sites define the position and timing of endosome fission. Cell.

[113] Almeida C., Amaral M.D. (2020). A central role of the endoplasmic reticulum in the cell emerges from its functional contact sites with multiple organelles. Cell. Mol. Life Sci..

[114] Scorrano L., De Matteis M.A., Emr S., Giordano F., Hajnóczky G., Kornmann B., Lackner L.L., Levine T.P., Pellegrini L., Reinisch K. (2019). Coming together to define membrane contact sites. Nat. Commun..

[115] Elbaz Y., Schuldiner M. (2011). Staying in touch: The molecular era of organelle contact sites. Trends Biochem. Sci..

[116] Phillips M.J., Voeltz G.K. (2016). Structure and function of ER membrane contact sites with other organelles. Nat. Rev. Mol. Cell Biol..

[117] Lahiri S., Toulmay A., Prinz W.A. (2015). Membrane contact sites, gateways for lipid homeostasis. Curr. Opin. Cell Biol..

[118] Rowland A.A., Voeltz G.K. (2012). Endoplasmic reticulum-mitochondria contacts: Function of the junction. Nat. Rev. Mol. Cell Biol..

[119] Morel E. (2020). Endoplasmic Reticulum Membrane and Contact Site Dynamics in Autophagy Regulation and Stress Response. Front. Cell Dev. Biol..

[120] Rieusset J. (2018). The role of endoplasmic reticulum-mitochondria contact sites in the control of glucose homeostasis: An update. Cell Death Dis..

[121] Csordás G., Renken C., Várnai P., Walter L., Weaver D., Buttle K.F., Balla T., Mannella C.A., Hajnóczky G. (2006). Structural and functional features and significance of the physical linkage between ER and mitochondria. J. Cell Biol..

[122] Shore G.C., Tata J.R. (1977). Two fractions of rough endoplasmic reticulum from rat liver. I. Recovery of rapidly sedimenting endoplasmic reticulum in association with mitochondria. J. Cell Biol..

[123] Csordás G., Weaver D., Hajnóczky G. (2018). Endoplasmic Reticulum-Mitochondrial Contactology: Structure and Signaling Functions. Trends Cell Biol..

[124] Wang W.X., Prajapati P., Nelson P.T., Springer J.E. (2020). The Mitochondria-Associated ER Membranes Are Novel Subcellular Locations Enriched for Inflammatory-Responsive MicroRNAs. Mol. Neurobiol..

[125] Gao Q., Goodman J.M. (2015). The lipid droplet-a well-connected organelle. Front. Cell Dev. Biol..

[126] Olzmann J.A., Carvalho P. (2019). Dynamics and functions of lipid droplets. Nat. Rev. Mol. Cell Biol..

[127] Walther T.C., Chung J., Farese R.V. (2017). Lipid Droplet Biogenesis. Annu. Rev. Cell Dev. Biol..

[128] Wilfling F., Wang H., Haas J.T., Krahmer N., Gould T.J., Uchida A., Cheng J.X., Graham M., Christiano R., Fröhlich F. (2013). Triacylglycerol synthesis enzymes mediate lipid droplet growth by relocalizing from the ER to lipid droplets. Dev. Cell.

[129] Choudhary V., Golani G., Joshi A.S., Cottier S., Schneiter R., Prinz W.A., Kozlov M.M. (2018). Architecture of Lipid Droplets in Endoplasmic Reticulum Is Determined by Phospholipid Intrinsic Curvature. Curr. Biol..

[130] Kassan A., Herms A., Fernández-Vidal A., Bosch M., Schieber N.L., Reddy B.J., Fajardo A., Gelabert-Baldrich M., Tebar F., Enrich C. (2013). Acyl-CoA synthetase 3 promotes lipid droplet biogenesis in ER microdomains. J. Cell Biol..

[131] Santinho A., Salo V.T., Chorlay A., Li S., Zhou X., Omrane M., Ikonen E., Thiam A.R. (2020). Membrane Curvature Catalyzes Lipid Droplet Assembly. Curr. Biol..

[132] Salo V.T., Belevich I., Li S., Karhinen L., Vihinen H., Vigouroux C., Magré J., Thiele C., Hölttä-Vuori M., Jokitalo E. (2016). Seipin regulates ER-lipid droplet contacts and cargo delivery. EMBO J..

[133] Wang H., Becuwe M., Housden B.E., Chitraju C., Porras A.J., Graham M.M., Liu X.N., Thiam A.R., Savage D.B., Agarwal A.K. (2016). Seipin is required for converting nascent to mature lipid droplets. eLife.

[134] Datta S., Liu Y., Hariri H., Bowerman J., Henne W.M. (2019). Cerebellar ataxia disease-associated Snx14 promotes lipid droplet growth at ER-droplet contacts. J. Cell Biol..

[135] Li D., Zhao Y.G., Li D., Zhao H., Huang J., Miao G., Feng D., Liu P., Li D., Zhang H. (2019). The ER-Localized Protein DFCP1 Modulates ER-Lipid Droplet Contact Formation. Cell Rep..

[136] Xu D., Li Y., Wu L., Li Y., Zhao D., Yu J., Huang T., Ferguson C., Parton R.G., Yang H. (2018). Rab18 promotes lipid droplet (LD) growth by tethering the ER to LDs through SNARE and NRZ interactions. J. Cell Biol..

[137] Ebihara C., Ebihara K., Aizawa-Abe M., Mashimo T., Tomita T., Zhao M., Gumbilai V., Kusakabe T., Yamamoto Y., Aotani D. (2015). Seipin is necessary for normal brain development and spermatogenesis in addition to adipogenesis. Hum. Mol. Genet..

[138] Huang H.S., Yoon B.J., Brooks S., Bakal R., Berrios J., Larsen R.S., Wallace M.L., Han J.E., Chung E.H., Zylka M.J. (2014). Snx14 regulates neuronal excitability, promotes synaptic transmission, and is imprinted in the brain of mice. PLoS ONE.

[139] Nian F.S., Li L.L., Cheng C.Y., Wu P.C., Lin Y.T., Tang C.Y., Ren B.S., Tai C.Y., Fann M.J., Kao L.S. (2019). Rab18 Collaborates with Rab7 to Modulate Lysosomal and Autophagy Activities in the Nervous System: An Overlapping Mechanism for Warburg Micro Syndrome and Charcot-Marie-Tooth Neuropathy Type 2B. Mol. Neurobiol..

[140] Wang L., Hong J., Wu Y., Liu G., Yu W., Chen L. (2018). Seipin deficiency in mice causes loss of dopaminergic neurons via aggregation and phosphorylation of α-synuclein and neuroinflammation. Cell Death Dis..

[141] Parton R.G., Bosch M., Steiner B., Pol A. (2020). Novel contact sites between lipid droplets, early endosomes, and the endoplasmic reticulum. J. Lipid Res..

[142] Saheki Y., De Camilli P. (2017). Endoplasmic Reticulum-Plasma Membrane Contact Sites. Annu. Rev. Biochem..

[143] Orci L., Ravazzola M., Le Coadic M., Shen W.W., Demaurex N., Cosson P. (2009). From the Cover: STIM1-induced precortical and cortical subdomains of the endoplasmic reticulum. Proc. Natl. Acad. Sci. USA.

[144] Palade G.E. (1956). The endoplasmic reticulum. J. Biophys. Biochem. Cytol..

[145] Porter K.R., Palade G.E. (1957). Studies on the endoplasmic reticulum. III. Its form and distribution in striated muscle cells. J. Biophys. Biochem. Cytol..

[146] Machaca K. (2020). Ca^2+^ signaling and lipid transfer ‘pas a deux’ at ER-PM contact sites orchestrate cell migration. Cell Calcium.

[147] Friedman R. (2020). Measurements of neuronal morphological variation across the rat neocortex. Neurosci. Lett..

[148] Broadwell R.D., Cataldo A.M. (1983). The neuronal endoplasmic reticulum: Its cytochemistry and contribution to the endomembrane system. I. Cell bodies and dendrites. J. Histochem. Cytochem..

[149] Palade G.E., Porter K.R. (1954). Studies on the endoplasmic reticulum. I. Its identification in cells in situ. J. Exp. Med..

[150] Beaudet A., Rambourg A. (1983). The tridimensional structure of Nissl bodies: A stereoscopic study in ventral horn cells of rat spinal cord. Anat. Rec..

[151] Nievel J.G., Cumings J.N. (1967). Nissl substance and ribosomal aggregates. Nature.

[152] Moon L.D.F. (2018). Chromatolysis: Do injured axons regenerate poorly when ribonucleases attack rough endoplasmic reticulum, ribosomes and RNA?. Dev. Neurobiol..

[153] Boorman G. (2015). Boorman’s Pathology of the Rat.

[154] Oyanagi K., Yamazaki M., Takahashi H., Watabe K., Wada M., Komori T., Morita T., Mizutani T. (2008). Spinal anterior horn cells in sporadic amyotrophic lateral sclerosis show ribosomal detachment from, and cisternal distention of the rough endoplasmic reticulum. Neuropathol. Appl. Neurobiol..

[155] Dziewulska D., Gogol A., Gogol P., Rafalowska J. (2013). Enlargement of the Nissl substance as a manifestation of early damage to spinal cord motoneurons in amyotrophic lateral sclerosis. Clin. Neuropathol..

[156] Tao-Cheng J.H. (2018). Activity-dependent decrease in contact areas between subsurface cisterns and plasma membrane of hippocampal neurons. Mol. Brain.

[157] Palay S.L., Sotelo C., Peters A., Orkand P.M. (1968). The axon hillock and the initial segment. J. Cell Biol..

[158] Lindsey J.D., Ellisman M.H. (1985). The neuronal endomembrane system. III. The origins of the axoplasmic reticulum and discrete axonal cisternae at the axon hillock. J. Neurosci..

[159] Kosaka T. (1980). The axon initial segment as a synaptic site: Ultrastructure and synaptology of the initial segment of the pyramidal cell in the rat hippocampus (CA3 region). J. Neurocytol..

[160] Peters A., Proskauer C.C., Kaiserman-Abramof I.R. (1968). The small pyramidal neuron of the rat cerebral cortex. The axon hillock and initial segment. J. Cell Biol..

[161] Broadwell R.D., Cataldo A.M. (1984). The neuronal endoplasmic reticulum: Its cytochemistry and contribution to the endomembrane system. II. Axons and terminals. J. Comp. Neurol..

[162] Droz B., Rambourg A., Koenig H.L. (1975). The smooth endoplasmic reticulum: Structure and role in the renewal of axonal membrane and synaptic vesicles by fast axonal transport. Brain Res..

[163] Yalçın B., Zhao L., Stofanko M., O’Sullivan N.C., Kang Z.H., Roost A., Thomas M.R., Zaessinger S., Blard O., Patto A.L. (2017). Modeling of axonal endoplasmic reticulum network by spastic paraplegia proteins. eLife.

[164] Terasaki M. (2018). Axonal endoplasmic reticulum is very narrow. J. Cell Sci..

[165] Dailey M.E., Bridgman P.C. (1989). Dynamics of the endoplasmic reticulum and other membranous organelles in growth cones of cultured neurons. J. Neurosci..

[166] Dotti C.G., Sullivan C.A., Banker G.A. (1988). The establishment of polarity by hippocampal neurons in culture. J. Neurosci..

[167] Funahashi Y., Namba T., Nakamuta S., Kaibuchi K. (2014). Neuronal polarization in vivo: Growing in a complex environment. Curr. Opin. Neurobiol..

[168] Lefebvre J.L., Sanes J.R., Kay J.N. (2015). Development of dendritic form and function. Annu. Rev. Cell Dev. Biol..

[169] Ramírez O.A., Härtel S., Couve A. (2011). Location matters: The endoplasmic reticulum and protein trafficking in dendrites. Biol. Res..

[170] Chen C.C., Lu J., Zuo Y. (2014). Spatiotemporal dynamics of dendritic spines in the living brain. Front. Neuroanat..

[171] Frank A.C., Huang S., Zhou M., Gdalyahu A., Kastellakis G., Silva T.K., Lu E., Wen X., Poirazi P., Trachtenberg J.T. (2018). Hotspots of dendritic spine turnover facilitate clustered spine addition and learning and memory. Nat. Commun..

[172] Ratliff W.A., Delic V., Pick C.G., Citron B.A. (2020). Dendritic arbor complexity and spine density changes after repetitive mild traumatic brain injury and neuroprotective treatments. Brain Res..

[173] Segal M., Vlachos A., Korkotian E. (2010). The spine apparatus, synaptopodin, and dendritic spine plasticity. Neuroscientist.

[174] Fifkova E., Markham J.A., Delay R.J. (1983). Calcium in the spine apparatus of dendritic spines in the dentate molecular layer. Brain Res..

[175] Gray E.G. (1959). Axo-somatic and axo-dendritic synapses of the cerebral cortex: An electron microscope study. J. Anat..

[176] Deller T., Merten T., Roth S.U., Mundel P., Frotscher M. (2000). Actin-associated protein synaptopodin in the rat hippocampal formation: Localization in the spine neck and close association with the spine apparatus of principal neurons. J. Comp. Neurol..

[177] Harris K.M., Weinberg R.J. (2012). Ultrastructure of synapses in the mammalian brain. Cold Spring Harb. Perspect. Biol..

[178] Spacek J. (1985). Three-dimensional analysis of dendritic spines. II. Spine apparatus and other cytoplasmic components. Anat. Embryol..

[179] Spacek J., Harris K.M. (1997). Three-dimensional organization of smooth endoplasmic reticulum in hippocampal CA1 dendrites and dendritic spines of the immature and mature rat. J. Neurosci..

[180] Ng A.N., Doherty A.J., Lombroso P.J., Emptage N.J., Collingridge G.L. (2014). Rapid regulation of endoplasmic reticulum dynamics in dendritic spines by NMDA receptor activation. Mol. Brain.

[181] Perez-Alvarez A., Yin S., Schulze C., Hammer J.A., Wagner W., Oertner T.G. (2020). Endoplasmic reticulum visits highly active spines and prevents runaway potentiation of synapses. Nat. Commun..

[182] Cooney J.R., Hurlburt J.L., Selig D.K., Harris K.M., Fiala J.C. (2002). Endosomal compartments serve multiple hippocampal dendritic spines from a widespread rather than a local store of recycling membrane. J. Neurosci..

[183] Cui-Wang T., Hanus C., Cui T., Helton T., Bourne J., Watson D., Harris K.M., Ehlers M.D. (2012). Local zones of endoplasmic reticulum complexity confine cargo in neuronal dendrites. Cell.

[184] Viero G., Lunelli L., Passerini A., Bianchini P., Gilbert R.J., Bernabò P., Tebaldi T., Diaspro A., Pederzolli C., Quattrone A. (2015). Three distinct ribosome assemblies modulated by translation are the building blocks of polysomes. J. Cell Biol..

[185] Warner J.R., Rich A., Hall C.E. (1962). Electron Microscope Studies of Ribosomal Clusters Synthesizing Hemoglobin. Science.

[186] Rosenbluth J. (1962). Subsurface cisterns and their relationship to the neuronal plasma membrane. J. Cell Biol..

[187] Palay S.L., Chan-Palay V. (2012). Cerebellar Cortex: Cytology and Organization.

[188] Deardorff A.S., Romer S.H., Sonner P.M., Fyffe R.E. (2014). Swimming against the tide: Investigations of the C-bouton synapse. Front. Neural Circuits.

[189] Gallart-Palau X., Tarabal O., Casanovas A., Sábado J., Correa F.J., Hereu M., Piedrafita L., Calderó J., Esquerda J.E. (2014). Neuregulin-1 is concentrated in the postsynaptic subsurface cistern of C-bouton inputs to α-motoneurons and altered during motoneuron diseases. FASEB J..

[190] Witts E.C., Zagoraiou L., Miles G.B. (2014). Anatomy and function of cholinergic C bouton inputs to motor neurons. J. Anat..

[191] Öztürk Z., O’Kane C.J., Pérez-Moreno J.J. (2020). Axonal Endoplasmic Reticulum Dynamics and Its Roles in Neurodegeneration. Front. Neurosci..

[192] Menzies F.M., Fleming A., Caricasole A., Bento C.F., Andrews S.P., Ashkenazi A., Fullgrabe J., Jackson A., Jimenez Sanchez M., Karabiyik C. (2017). Autophagy and Neurodegeneration: Pathogenic Mechanisms and Therapeutic Opportunities. Neuron.

[193] Xu L., Wang X., Zhou J., Qiu Y., Shang W., Liu J.P., Wang L., Tong C. (2020). Miga-mediated endoplasmic reticulum-mitochondria contact sites regulate neuronal homeostasis. eLife.

[194] Shirokova O.M., Pchelin P.V., Mukhina I.V. (2020). MERCs. The Novel Assistant to Neurotransmission?. Front. Neurosci..

[195] Raeisossadati R., Ferrari M.F.R. (2020). Mitochondria-ER Tethering in Neurodegenerative Diseases. Cell Mol. Neurobiol..

[196] Leal N.S., Dentoni G., Schreiner B., Naia L., Piras A., Graff C., Cattaneo A., Meli G., Hamasaki M., Nilsson P. (2020). Amyloid Β-Peptide Increases Mitochondria-Endoplasmic Reticulum Contact Altering Mitochondrial Function and Autophagosome Formation in Alzheimer’s Disease-Related Models. Cells.

[197] Ferreira A., Cáceres A. (1989). The expression of acetylated microtubules during axonal and dendritic growth in cerebellar macroneurons which develop in vitro. Brain Res. Dev. Brain Res..

[198] Tabb J.S., Molyneaux B.J., Cohen D.L., Kuznetsov S.A., Langford G.M. (1998). Transport of ER vesicles on actin filaments in neurons by myosin V. J. Cell Sci..

[199] Wagner W., Brenowitz S.D., Hammer J.A. (2011). Myosin-Va transports the endoplasmic reticulum into the dendritic spines of Purkinje neurons. Nat. Cell Biol..

[200] Bailey A.P., Koster G., Guillermier C., Hirst E.M., MacRae J.I., Lechene C.P., Postle A.D., Gould A.P. (2015). Antioxidant Role for Lipid Droplets in a Stem Cell Niche of Drosophila. Cell.

[201] Inloes J.M., Hsu K.L., Dix M.M., Viader A., Masuda K., Takei T., Wood M.R., Cravatt B.F. (2014). The hereditary spastic paraplegia-related enzyme DDHD2 is a principal brain triglyceride lipase. Proc. Natl. Acad. Sci. USA.

[202] Liu L., Zhang K., Sandoval H., Yamamoto S., Jaiswal M., Sanz E., Li Z., Hui J., Graham B.H., Quintana A. (2015). Glial lipid droplets and ROS induced by mitochondrial defects promote neurodegeneration. Cell.

[203] Ioannou M.S., Jackson J., Sheu S.H., Chang C.L., Weigel A.V., Liu H., Pasolli H.A., Xu C.S., Pang S., Matthies D. (2019). Neuron-Astrocyte Metabolic Coupling Protects against Activity-Induced Fatty Acid Toxicity. Cell.

[204] Barber C.N., Raben D.M. (2019). Lipid Metabolism Crosstalk in the Brain: Glia and Neurons. Front Cell Neurosci..

[205] Rickman O.J., Baple E.L., Crosby A.H. (2020). Lipid metabolic pathways converge in motor neuron degenerative diseases. Brain.

[206] Windpassinger C., Auer-Grumbach M., Irobi J., Patel H., Petek E., Hörl G., Malli R., Reed J.A., Dierick I., Verpoorten N. (2004). Heterozygous missense mutations in BSCL2 are associated with distal hereditary motor neuropathy and Silver syndrome. Nat. Genet..

[207] Yagi T., Ito D., Nihei Y., Ishihara T., Suzuki N. (2011). N88S seipin mutant transgenic mice develop features of seipinopathy/BSCL2-related motor neuron disease via endoplasmic reticulum stress. Hum. Mol. Genet..

[208] Klemm R.W., Norton J.P., Cole R.A., Li C.S., Park S.H., Crane M.M., Li L., Jin D., Boye-Doe A., Liu T.Y. (2013). A conserved role for atlastin GTPases in regulating lipid droplet size. Cell Rep..

[209] Papadopoulos C., Orso G., Mancuso G., Herholz M., Gumeni S., Tadepalle N., Jüngst C., Tzschichholz A., Schauss A., Höning S. (2015). Spastin binds to lipid droplets and affects lipid metabolism. PLoS Genet..

[210] Renvoisé B., Malone B., Falgairolle M., Munasinghe J., Stadler J., Sibilla C., Park S.H., Blackstone C. (2016). Reep1 null mice reveal a converging role for hereditary spastic paraplegia proteins in lipid droplet regulation. Hum. Mol. Genet..

[211] Fanning S., Selkoe D., Dettmer U. (2020). Parkinson’s disease: Proteinopathy or lipidopathy?. NPJ Parkinson’s Dis..

[212] Licker V., Turck N., Kövari E., Burkhardt K., Côte M., Surini-Demiri M., Lobrinus J.A., Sanchez J.C., Burkhard P.R. (2014). Proteomic analysis of human substantia nigra identifies novel candidates involved in Parkinson’s disease pathogenesis. Proteomics.

[213] De Meyts P. (2004). Insulin and its receptor: Structure, function and evolution. Bioessays.

[214] Fu Z., Gilbert E.R., Liu D. (2013). Regulation of insulin synthesis and secretion and pancreatic Beta-cell dysfunction in diabetes. Curr. Diabetes Rev..

[215] Hafner A.S., Donlin-Asp P.G., Leitch B., Herzog E., Schuman E.M. (2019). Local protein synthesis is a ubiquitous feature of neuronal pre- and postsynaptic compartments. Science.

[216] Luarte A., Cornejo V.H., Bertin F., Gallardo J., Couve A. (2018). The axonal endoplasmic reticulum: One organelle-many functions in development, maintenance, and plasticity. Dev. Neurobiol..

[217] Martinez G., Duran-Aniotz C., Cabral-Miranda F., Vivar J.P., Hetz C. (2017). Endoplasmic reticulum proteostasis impairment in aging. Aging Cell.

[218] Bellucci A., Navarria L., Zaltieri M., Falarti E., Bodei S., Sigala S., Battistin L., Spillantini M., Missale C., Spano P. (2011). Induction of the unfolded protein response by alpha-synuclein in experimental models of Parkinson’s disease. J. Neurochem..

[219] Gorbatyuk M.S., Shabashvili A., Chen W., Meyers C., Sullivan L.F., Salganik M., Lin J.H., Lewin A.S., Muzyczka N., Gorbatyuk O.S. (2012). Glucose regulated protein 78 diminishes alpha-synuclein neurotoxicity in a rat model of Parkinson disease. Mol. Ther..

[220] Hetz C., Saxena S. (2017). ER stress and the unfolded protein response in neurodegeneration. Nat. Rev. Neurol..

[221] Dykstra K.M., Pokusa J.E., Suhan J., Lee T.H. (2010). Yip1A structures the mammalian endoplasmic reticulum. Mol. Biol. Cell.

[222] Schuck S., Gallagher C.M., Walter P. (2014). ER-phagy mediates selective degradation of endoplasmic reticulum independently of the core autophagy machinery. J. Cell Sci..

[223] Varadarajan S., Bampton E.T., Smalley J.L., Tanaka K., Caves R.E., Butterworth M., Wei J., Pellecchia M., Mitcheson J., Gant T.W. (2012). A novel cellular stress response characterised by a rapid reorganisation of membranes of the endoplasmic reticulum. Cell Death Differ..

[224] Sharoar M.G., Shi Q., Ge Y., He W., Hu X., Perry G., Zhu X., Yan R. (2016). Dysfunctional tubular endoplasmic reticulum constitutes a pathological feature of Alzheimer’s disease. Mol. Psychiatry.

[225] Mao Y., Chen X., Xu M., Fujita K., Motoki K., Sasabe T., Homma H., Murata M., Tagawa K., Tamura T. (2016). Targeting TEAD/YAP-transcription-dependent necrosis, TRIAD, ameliorates Huntington’s disease pathology. Hum. Mol. Genet..

[226] Nishimura A.L., Mitne-Neto M., Silva H.C., Richieri-Costa A., Middleton S., Cascio D., Kok F., Oliveira J.R., Gillingwater T., Webb J. (2004). A mutation in the vesicle-trafficking protein VAPB causes late-onset spinal muscular atrophy and amyotrophic lateral sclerosis. Am. J. Hum. Genet..

[227] Paillusson S., Gomez-Suaga P., Stoica R., Little D., Gissen P., Devine M.J., Noble W., Hanger D.P., Miller C.C.J. (2017). alpha-Synuclein binds to the ER-mitochondria tethering protein VAPB to disrupt Ca(2+) homeostasis and mitochondrial ATP production. Acta Neuropathol..

[228] Blackstone C. (2012). Cellular pathways of hereditary spastic paraplegia. Annu. Rev. Neurosci..

[229] Blackstone C. (2018). Hereditary spastic paraplegia. Handb. Clin. Neurol..

[230] Oliva M.K., Pérez-Moreno J.J., O’Shaughnessy J., Wardill T.J., O’Kane C.J. (2020). Endoplasmic Reticulum Lumenal Indicators in Drosophila Reveal Effects of HSP-Related Mutations on Endoplasmic Reticulum Calcium Dynamics. Front. Neurosci..

[231] De Gregorio C., Delgado R., Ibacache A., Sierralta J., Couve A. (2017). Drosophila Atlastin in motor neurons is required for locomotion and presynaptic function. J. Cell Sci..

[232] Summerville J.B., Faust J.F., Fan E., Pendin D., Daga A., Formella J., Stern M., McNew J.A. (2016). The effects of ER morphology on synaptic structure and function in Drosophila melanogaster. J. Cell Sci..

[233] Beetz C., Koch N., Khundadze M., Zimmer G., Nietzsche S., Hertel N., Huebner A.K., Mumtaz R., Schweizer M., Dirren E. (2013). A spastic paraplegia mouse model reveals REEP1-dependent ER shaping. J. Clin. Investig..

[234] Vila M., Ramonet D., Perier C. (2008). Mitochondrial alterations in Parkinson’s disease: New clues. J. Neurochem..

[235] Hoozemans J.J., van Haastert E.S., Nijholt D.A., Rozemuller A.J., Scheper W. (2012). Activation of the unfolded protein response is an early event in Alzheimer’s and Parkinson’s disease. Neurodegener. Dis..

[236] Shahmoradian S.H., Lewis A.J., Genoud C., Hench J., Moors T.E., Navarro P.P., Castano-Diez D., Schweighauser G., Graff-Meyer A., Goldie K.N. (2019). Lewy pathology in Parkinson’s disease consists of crowded organelles and lipid membranes. Nat. Neurosci..

[237] Airavaara M., Parkkinen I., Konovalova J., Albert K., Chmielarz P., Domanskyi A. (2020). Back and to the Future: From Neurotoxin-Induced to Human Parkinson’s Disease Models. Curr. Protoc. Neurosci..

[238] Ryu E.J., Harding H.P., Angelastro J.M., Vitolo O.V., Ron D., Greene L.A. (2002). Endoplasmic reticulum stress and the unfolded protein response in cellular models of Parkinson’s disease. J. Neurosci..

[239] Rivero-Rios P., Gomez-Suaga P., Fdez E., Hilfiker S. (2014). Upstream deregulation of calcium signaling in Parkinson’s disease. Front. Mol. Neurosci..

[240] Surmeier D.J., Schumacker P.T., Guzman J.D., Ilijic E., Yang B., Zampese E. (2017). Calcium and Parkinson’s disease. Biochem. Biophys. Res. Commun..

[241] Mamelak M. (2018). Parkinson’s Disease, the Dopaminergic Neuron and Gammahydroxybutyrate. Neurol. Ther..

[242] Venda L.L., Cragg S.J., Buchman V.L., Wade-Martins R. (2010). alpha-Synuclein and dopamine at the crossroads of Parkinson’s disease. Trends Neurosci..

[243] Cooper A.A., Gitler A.D., Cashikar A., Haynes C.M., Hill K.J., Bhullar B., Liu K., Xu K., Strathearn K.E., Liu F. (2006). Alpha-synuclein blocks ER-Golgi traffic and Rab1 rescues neuron loss in Parkinson’s models. Science.

[244] Credle J.J., Forcelli P.A., Delannoy M., Oaks A.W., Permaul E., Berry D.L., Duka V., Wills J., Sidhu A. (2015). alpha-Synuclein-mediated inhibition of ATF6 processing into COPII vesicles disrupts UPR signaling in Parkinson’s disease. Neurobiol. Dis..

[245] Hübner C.A., Kurth I. (2014). Membrane-shaping disorders: A common pathway in axon degeneration. Brain.

[246] Zhang H., Hu J. (2016). Shaping the Endoplasmic Reticulum into a Social Network. Trends Cell Biol..

[247] Bernard-Marissal N., Chrast R., Schneider B.L. (2018). Endoplasmic reticulum and mitochondria in diseases of motor and sensory neurons: A broken relationship?. Cell Death Dis..

